# Supplementary material for: Ubinuclein 2 is essential for mouse development and functions in X chromosome inactivation
Source: PLoS Genet. 2025 Jun 2;21(6):e1011711. doi: 10.1371/journal.pgen.1011711 (PMC12165345; doi:10.1371/journal.pgen.1011711)
Supplement: S1 Text — (PDF) [file pgen.1011711.s001.pdf]

Supplementary Text

for

Ubinuclein 2 is essential for mouse development and functions in X chromosome inactivation

Asun Monfort, Giulio Di Minin, Sarah Sting, Charles Etienne Dumeau,

Peter Scambler & Anton Wutz

Extended Materials and Methods

gRNA design for the CRISPR/Cas nucleases

Gene specific gRNAs were designed using the CHOPCHOP (Labun et al. 2016), GuideScan (Perez et al. 2017), and E-CRISPR (Heigwer et al. 2014) prediction programs. Sequences are listed in Suppl. Table 5.

Gene editing with Cas9 expression vectors

gRNAs were cloned into pX458 (Addgene, #48138). 2µg of the vector were transfected into 300000 cells using Lipofectamin 2000 (ThermoFisher) over-night. 36 hours later, the GFP positive population was sorted on a high-speed fluorescence-activated cell sorter (Beckman Coulter MoFlo Astrios) and individual cells were grown in 96-wells. Gene specific deletions and insertions were identified by PCR and Sanger sequencing. For introducing an HA tag into *Ubn2*, 1µg of ssDNA donor template (5'-tttcttttctcagatggaggccaaagtaaaggggacactaagttaccacggaaacctcagTCCGCCTGGTCCCACCCTCAGTTTG AGAAAGGCGGCGGCTACCCCTACGACGTGCCCGACTACGCCTGActttccagcaagggggagaggaaccacttggctggct ggcgggaccgacctgatgggaag-3'), and 2µg of Cas9 vector were co-transfected into ESCs as described above.

Gene editing with AsCas12a ribonucleoproteins (RNPs)

Synthetic crRNA oligos were purchased from Integrated DNA Technologies (IDT). Briefly, 10^6 ESCs in Opti-MEM (ThermoFisher, #31985062) were mixed with pre-assembled AsCas12a RNP complexes in Cas12a buffer (8 mM TrisHCl pH7.4, 0.1 mM EDTA, 2 mM $MgCl_2$). Subsequently, electroporation was carried out using a NEPA21 electroporator (Nepa Gene) in a final volume of 100µl as reported previously (Kissling et al. 2019). 12 hours later GFP expressing cells were sorted and deposited at clonal density into 96-wells. Mutations were identified by PCR (S5 Table).

Gene editing in mice with Cas9 RNPs

For zygote injection, independent or chimeric crRNA/tracrRNA nucleotides were kindly provided by Synthego, or purchased from Integrated DNA Technology, and Microsynth AG. Briefly, 20 pmol Cas9 were mixed with 40 pmol of sgRNA in injection buffer (8 mM TrisHCl pH 7.4, 0.1 mM EDTA) adjusted to a final volume of 12.5 µl and used for pronuclear microinjection of C57BL/6 zygotes by the ETH transgenics service. To obtain mutant mice, injected embryos were transferred to pseudo-pregnant recipients. Mutations were identified by PCR using DNA from ear biopsies from founder mice. Cas9 RNP nuclease activity was confirmed by *in vitro* cleavage assays. Briefly, 5 µg plasmid containing the target sequence were incubated with pre-assembled Cas9 RNP complexes (200 pmol Cas9 and 400 pmol of crRNA) in 1X Cutsmart NEB buffer in a final volume of 130 µl over-night at 37 ^{o}C. After digestion with 2 µl of proteinase K (20mg/ml) for 30 min at 55 ^{o}C, cleavage products were analyzed by gel electrophoresis.

Nuclei isolation from liver and Western Blot

Nuclear isolation from mouse liver was performed as described previously (Nagata et al., 2009). Briefly, to remove blood from livers, livers were injected with 150 µL of heparin in 200 µL of PBS, followed by 15 mL of ice-cold PBS. To get the liver cells into suspension, 0.5 g of liver was passed through a 40 µM pore size cells strainer into chilled buffer A (250 mM sucrose, 5 mM $MgCl_2$, 10 mM Tris-Hcl (pH 7.4)). To pellet the nuclei, the resulting suspension of disrupted cells was centrifuged, washed once with chilled buffer A and subsequently resuspended in 9 volumes of ice-cold buffer B (2.0 M sucrose, 1 mM $MgCl_2$, 10 mM Tris-HCl (pH 7.4)). Nuclei were then spun down at 70'000g for 80 minutes and the upper layer discarded. The white pellet of isolated nuclei was then resuspended in 300 µL of RIPA buffer (50 mM TrisHCl pH 8, 150 mM NaCl, 1 mM EDTA, 1% NP40, 0.25% Deoxycholate). Protein concentrations were quantified using the Bio-Rad DC Protein Assay (DC Protein Assay Kit II, Bio-Rad, 5000112) and resuspended in Laemmli sample buffer. Equal protein concentrations were subjected to SDS-PAGE. For detection of Ubn1, the anti-mouse Ubn-1 (Santa Cruz Biotechnology, sc-515340, 1:1000) was used. As

loading control, EZH2 was detected using the anti-mouse EZH2 mAb (Cell Signaling Technology #3147, 1:1000).

ESC culture, *Xist* induction and cell survival assay

HATX3 and HATX8 ESC lines and culture conditions were described previously (Monfort et al. 2015). *Xist* was induced by adding 1 µg/ml doxycycline to the culture medium. For sensitive detection of *Xist* function a clonal survival assay was developed. Briefly, ESCs were deposited into 96 well plates at single cell density and cultured for 5 to 6 days with or without *Xist* induction. Survival was assessed by colony counts and size binned in three categories (big, medium, and small) relative to the no Dox condition S2E Fig).

RNA isolation and expression analysis by quantitative RT-qPCR

Total RNA was isolated from ESCs using RNeasy Mini Kit (Quiagen, #74104) and DNA was removed using on-column DNAse I digestion (Qiagen, #79254). cDNA for real time PCR was synthesized from 500 ng total RNA in a 10 µl reaction using the PrimeScript RT Master Mix kit (Takara, #RR036A). Real-time quantitative PCR reactions were performed using SYBER Green and a LightCycler 480 system (Roche). Expression was normalized to *Eif4a2, Actin, Sdha*, and *Rrm12* expression levels. Primer sequences are listed in S5 Table.

Cloning of *Hira* and *Ubn2* expression vectors and transfection

First-strand cDNA was synthesized with SuperScript III from poly(A)-selected RNA primed with oligo(dT) (Invitrogen, #18080-051). The *Hira* coding region was amplified by PCR using Phusion High-Fidelity DNA polymerase (NEB, #M0530S) and the primers 5'-atcatcatttaaatatgaagctcttgaagccaacc-3' and 5'-attattgcggccgcctacttgtctctcaggatgtccag-3'. The PCR product was cloned into a PiggyBac (Pb) vector containing an EF1-alpha promoter and a Ruby fluorescence reporter (System Biosciences, Cat No. PB531A-2). The *Ubn2* cDNA (Dharmacon, #MMM1013-202770327) was cloned into the PiggyBac

vector described above. The ΔBLLF1 mutation was introduced by cutting the *Ubn2* expression vector with two Cas9 RNPs using the gRNAs *Ubn2* BLLF left and *Ubn2* BLLF right (Suppl. Table 5). Subsequently, gel purified vector fragment was circularized using ligase. 300000 ESCs were transfected with 3 µg of *Hira*, *Ubn2*, or ΔBLLF1 expression vector and 1 µg of PiggyBac-transposase vector using Lipofectamin 2000 (Invitrogen). 36 hours later, Ruby positive ESCs were isolated by cell sorting individual cells into 96-wells. After two passages ESC clones were resorted for stable Ruby expression.

Subcellular protein fractionation

For nuclear protein fractionation 10 confluent 150 cm^2 plates of ESCs were used. All the buffers contained a mix of protease and phosphatase inhibitors: AEBSF (Sigma, #A8456) or PMSF (Sigma, #P7626**),** Trypsin inhibitor (Roche, #10109886001), PepA (Sigma, #P-4265), Antipain (Sigma, #A-6191), Aprotinin (Roche, #1-583794), Benzamidine HCL (Sigma, #B-6506), Leupeptin (Roche, #1-034626), NaF (Sigma, #S7920), Na3VO4 (Sigma, #S6508), NaPPi (Sigma, #221368). Cells were washed twice with PBS, scraped off the plates, and pelleted by centrifugation in an Eppendorf tube for 10 minutes at 14000 rpm. Pellets were resuspended in 1 ml hypotonic buffer A (10 mM Hepes pH 7.6, 1.5 mM MgCl2, 10mM KCl), per plate, and incubated on ice for 15 min. Subsequently, the membranes were disrupted by homogenizing 10 times with a loose pestle in a 7 ml Dounce homogenizer. After adding NP40 to a final 0.5% concentration, samples were placed on a rotating wheel at 4 °C for 10 minutes. Nuclei were pelleted at 14000 rpm 10 min at 4 °C, and resuspended in high salt buffer C (Hepes pH 7.6 20 mM, Glycerol 20% (v/v), NaCl 420 mM, MgCl2 1.5 mM, EDTA 0.2 mM). 10 µl of nuclei suspension were checked for integrity under the microscope in a 1 : 1 Trypan blue solution before nuclear membranes were disrupted by 20 strokes with a loose pestle. Nuclear lysates were incubated on ice 15 min, rotated for 30 min on a wheel at 4 °C, and centrifuged at 14000 rpm 20 min 4°C. The pellets, containing chromatin and chromatin-bound proteins, were resuspended in 1ml modified RIPA buffer, (50 mM TrisHCl pH 8, 150 mM NaCl, 1 mM EDTA, 1% NP40, 0.25% Deoxycholate) incubated on ice 10 min,

rotated on a wheel 20 min at 4 °C, and centrifuged at 14000 rpm 10 min 4 °C. The resulting supernatant was the fraction enriched for HIRA and UBN2.

Co-Immunoprecipitation

2 mg of lysate were incubated over night at 4 °C on a rotator with 250U of Benzonase nuclease (Sigma-Aldrich, #E1014) and 55 µl (dry volume) of pre-washed anti-HA affinity matrix (Roche, #11815016001) in a final volume of 1 ml RIPA buffer. Subsequently, beads were washed twice for 5 min under rotation with wash buffer (50 mM TrisHCl pH 8, 150 mM NaCl, 1 mM EDTA, 0.02% NP40), and once with PBS. Proteins were eluted from the beads by resuspending in 2 x Laemmli buffer and boiling for 5 min at 95 °C.

SILAC labelling and proteomics analysis

ESCs were grown in SILAC medium for at least 10 passages to ensure full incorporation of the heavy labelled amino-acids. The customized Ndiff medium (without Arginine and Lysine; Stem Cell Sciences) was supplemented with 1000 units/ml mLIF, 3 µM Chiron 99021 (Axon, #1386), 1 µM PD 0325901 (Axon, #1408), 1X Pen/Strep (Gibco, #15140-122), 0.5% BSA FV-7.5% (Gibco, #15260-037), and $^{13}C_6{}^{15}N_4$ L-arginine and $^{13}C_6{}^{15}N_2$ l-lysine (#CNLM-539-H; #CNLM-291-H; #ULM-8766; #ULM-8347; Cambridge Isotope Laboratories). The cells were tested for the absence of ARG to PRO conversion.

Mass Spectrometry

WT HATX8 and HATX8-*Ubn2*-HA ESCs were grown in heavy and light SILAC medium respectively before sub-cellular fractionation and Co-IP were performed as described above. Washed, dry bead pellets containing the immunoprecipitated proteins were deposited in the proteomics service at the Functional Genomics Center Zürich (FGCZ) where samples were prepared for MS. Briefly, the HEAVY_WT and LIGHT_HA samples were mixed 1:1. Beads were washed once with 50 µl buffer (10 mM Tris/2 mM CaCl2, pH 8.2) and the supernatant removed. Washed beads were incubated in 45 µl

buffer (10 mM Tris/2 mM CaCl2, pH 8.2) + 5 µl trypsin (100 ng/µl in 10 mM HCl) for 30 min at 60°C using the Microwave generator (CEM Discover). Supernatant was collected and beads were washed once with 150 µl solvent (0.1% TFA/50% acetonitrile). Supernatants were combined, dried by vacuum concentration and dissolved in 20 µl 0.1% formic acid. Dissolved peptides were analyzed by LC-MS/MS.

Immunofluorescence analysis

For immunofluorescence staining cells were grown on multiwell Roboz Slides (Cellpoint Scientific, USA), and then fixed with 4% PFA 10 min, permeabilized in 3mM Na Citrate / 0.5% Triton X-100 for 10 min and unspecific binding was blocked with blocking buffer (PBS / 2.5% BSA / 0.1% Tween-20) for 30min at RT. Primary antibodies diluted in blocking buffer were incubated over night at 4 °C followed by incubation with secondary antibodies for 1h at RT. Nuclei were counterstained with 1.4 µM DAPI (Molecular Probes, #D1306). After several washes in PBS / 0.1% Tween-20 (PBST) slides were mounted using Vectashield (Reactolab, #H1000), or Mowiol (Sigma-Aldrich, #81381).

Combined immunofluorescence and *Xist* RNA FISH

Multiwell Roboz slides (Cellpoint Scientific, USA) were pre-coated over night with 5 µg/ml laminin in PBS (Corning, #354232) and 10^4 cells were plated per well. After 24h, the samples were rinsed once with PBS and fixed in freshly prepared 4% paraformaldehyde in PBS for 10 min at room temperature. After 2 washes with PBS, cells were permeabilized on ice for 5 min with 0.1% sodium citrate / 0.5% Triton X-100, washed twice in PBST, and blocked 30 min with PBST / 2.5% BSA. The slides were incubated for 45 min at room temperature with the primary antibody, and 45 min with the secondary antibody blocking buffer containing RiboLock RNAse inhibitor (Thermo Scientific, #EO0381). Slides were washed three times in PBST after each antibody incubation. Slides were post fixed with 4% paraformaldehyde in PBS for 10 min at room temperature, washed once with PBS, and twice in 2 x SSC (300 mM NaCl, 30 mM sodium citrate pH 7.4). The samples were air dried for 10 min at room temperature. Cy3-labeled *Xist* RNA FISH probe (Cy3 dCTP, GE Healthcare Amersham) was generated

by random priming with Prime-it II, (Stratagen, #300385) and repetitive elements were competed with mouse Cot-1 DNA (Invitrogen, #18440-016), salmon sperm DNA (Invitrogen, #15632-011) and tRNA (Invitrogen, #AM7119). The FISH probe was precipitated by adding 0.1 volumes 3M NaOAc pH 5.2 and 2.5 volumes 100% ethanol and centrifugation for 20 min full speed in an Eppendorf centrifuge, resuspended in 80 µl Hybrisol VII (MP Biomedicals), and denatured for 10 minutes at 74°C. 3 µl *Xist* probe diluted 1:3 in Hybrisol VII were applied to each well and incubated over night at 37 °C in a light protected humidified chamber. Slides were washed three times 5 min with 2 x SSC / 50% formamide at 39 °C, three more times 5 min with 2 x SSC at 39 °C, once 10 min with 1 x SSC at room temperature, and once with 4 x SSC at room temperature. Nuclei were counterstained with a 1.4 µM DAPI solution (Molecular Probes, #D1306) in 4 x SSC / 0.1% Tween-20 for 5 minutes.

Chromosome spreads and X chromosome paint

Cells were harvested and incubated in hypotonic medium (50% cell culture medium, 50% deionized water) for 10 minutes at 37°C. They were then fixed in methanol/acetic acid (3:1 ratio) and dropped onto glass slides. For staining of the X chromosome, XMP X green probe (MetaSystems D-1420-050-FI) was used according to manufaturer's instructions. Slides were mounted using Vectashield® Antifade Mounting Medium with Dapi (Vector Laboratories, H-1200-10).

Genotyping of ear biopsies

For DNA extraction, samples were lysed with 1X Ten9 / 1% SDS / 0.5 mg/ml proteinase K over-night, proteins precipitated with saturated NaCl, and DNA precipitated with isopropanol. The mice were genotyped by PCR. ThermoPol Taq DNA polymerase (NEB, #M0267L) was used to validate the *Ubn1* and *Ubn2* deletions, and Phusion High-Fidelity DNA polymerase (NEB, #M0530L) was used to genotype the *Hira-Neo* mutants. 100 ng of DNA was used per reaction. Primer sequences are listed in S5 Table.

Isolation of MEF from E11.5 embryos

Embryos were carefully dissected from maternal tissue and mechanically disaggregated by forcing 10 times through a 20G needle in 200 µl of Trypsin/EDTA solution. The disaggregated tissue suspension was neutralized with 3ml MEF medium (DMEM high glucose (Gibco, #41965-039), 1 x Pen/Strep (Gibco, #15140-122), 1 x NEAA (Gibco, #11140-035), 1 x L-Glutamine (Gibco, #25030-081), 1 x Sodium Pyruvate (Gibco, #11360-039), 4µl per 500 ml β-mercaptoethanol (Sigma, #M6250)) supplemented with 10% FBS, and incubated for 2 to 3 days in a 6 well plate. Thereafter cells were passaged as necessary.

Native ChIP

12 million ESCs were used as starting material and were divided in three immunoprecipitation experiments with different antibodies (4 million per ChIP). All the buffers were complemented with Complete Mini protease inhibitor (Roche, #11836153001) and 5 mM Na-butyrate deacetylase inhibitor. Pellets from of 12 million ESCs were washed with PBS 1X and spun. The dry cell pellets were resuspended in 90 µl of Lysis Buffer (LB) (50 mM TrisHCl pH 7.5, 150 mM NaCl, 0.1% sodium deoxycholate, 1% Triton, 5 mM CaCl2) and incubated 10 min on ice. Then, chromatin was digested by the addition of 60 µl of LB containing 0.3 µl Micrococcal nuclease (Cell signalling, #10011S) and incubation at 37 ^{o}C, 15 min with agitation. The reaction was stopped with 15 µl 0.5 M EDTA and pelleted at maximum speed in an Eppendorf centrifuge. The supernatant was mixed with an equal volume of Stop Buffer (SB) (50 mM TrisHCl pH 7.5, 150 mM NaCl, 0.1% sodium deoxycholate, 1% Triton, 30 mM EGTA, 30 mM EDTA) and the resulting chromatin preparation was diluted to 1 ml with LB / SB (1:1). 30 µl were separated for input, mixed with 150 µl of Elution Buffer (EB) (20 mM HEPES, 1mM EDTA, 0.5% SDS) containing 2 µl of proteinase K (20mg/ml) and incubated for 2h at 56 ^{o}C with agitation. DNA was then column purified (Qiagen, #28004), and eluted in 50 µl. 2 µl were loaded per q-PCR reaction. The 970 µl of chromatin solution were divided in three independent aliquots of 300 µl, filled up to 1ml with LB / SB (1:1) and incubated o/n, 4 ^{o}C on a wheel, with the corresponding antibody (S6

Table). The day after, unspecific binding of Dynabeads protein G (ThermoFisher, #10004D) was blocked by washing beads twice for 5 min with blocking buffer (PBS 1X, 0.5% Tween, 0.5% BSA). 15 µl of blocked Dynabeads G were added per tube and incubated 2h, 4 °C on a wheel. After removal of the supernatant, the beads were washed 4 times with Low salt buffer (20 mM TrisHCl pH 8, 150 mM NaCl, 0.1% sodium deoxycholate, 1% Triton, 0.1% SDS) , followed by 2 times with high salt buffer (20 mM TrisHCl pH 8, 360 mM NaCl, 0.1% sodium deoxycholate, 1% Triton, 0.1% SDS), 2 times LiCl buffer (10 mM TrisHCl pH 8, 0.25 M LiCl, 1% NP40, 1.1% sodium deoxycholate, 1mM EDTA) and 1 final wash with TE buffer pH 8. For H3K4me3 ChIP, beads were washed 8 times with low salt buffer and once with TE buffer pH 8. The immuno-precipitated material was eluted from beads by two consecutive incubations with 100 µl of EB + proteinase K for 15min, 56 °C, in an Eppendorf shaker at maximum speed. 200 µl eluate were obtained per tube and incubated for another 2h at 56 °C under agitation. Finally, the DNA was purified using the MinElute PCR purification kit (Qiagen, #28004), and eluted in 50 µl. 2 µl were used per q-PCR reaction. Primers are listed in S5 Table.

Native ChIP-Seq

ChIP-Seq was performed in triplicates for *Xist* inducible WT and Δ*Ubn1/Ubn2* ESCs, treated and non-treated with Dox for 48h. The protocol was followed as described for ChIP but chromatin was subjected to 20min digestion time with micrococcal nuclease. NGS library preparation and sequencing was performed by the sequencing service at the Functional Genomics Center Zürich (FGCZ). Briefly, 1 ng of ChIP-ed DNA was used for library preparation using the NEBNext ultra II DNA Library Prep Kit (New England Biolabs, Ipswich (NEB), #7103). The libraries were size-selected for fragments in the range of 300 to 600 bp with Agencourt AMPure XP magnetic beads (Beckman Coulter) before they were loaded into an Illumina HiSeq 2500 machine and 125 bp single-end sequencing was performed.

RNA-Seq transcriptome analysis

RNA-seq was performed in triplicates for *Xist* inducible WT, Δ*Ubn1,* Δ*Ubn2,* Δ*Ubn1/Ubn2,* and Δ*Hira* ESCs non-treated and treated with Dox for 48h. Total RNA was isolated from ESCs using RNeasy Mini Kit (Quiagen, #74104) and DNA was removed using on-column DNAse I digestion (Qiagen, #79254). NGS library preparation and sequencing was performed by the sequencing service at the Functional Genomics Center Zürich (FGCZ). Libraries were prepared with the Illumina TruSeq Stranded mRNA kit. Briefly, poly-adenylated RNA isolated using Oligo(dT) beads was reverse-transcribed into double-stranded cDNA before ligation of Truseq adapters. The Novaseq 6000 (Illumina, Inc, California, USA) was used for cluster generation and single-end 100 bp sequencing. Bioinformatics was performed by the FGCZ as a service.

CUT&RUN analysis

CUT&RUN was performed using CUT&RUN Assay Kit (Cell SignalingTechnology #86652) and antibodies against H3K27ac (Cell Signaling Technology #8173, 1:50) and H3K4me3 (Cell Signaling Technology rabbit mAb #9751, 1:50). DNA was purified from enriched chromatin samples using Spin Columns (Cell Signaling Technology #14209). For library prep, NEBNext Ultra II DNA Library Prep Kit for Illumina (NEB #E7103) was used. Libraries were sequenced on Illumina Miseq or Illumina NextSeq 2000.

ATACseq

ATACseq was performed in triplicates for *Xist* inducible WT and Δ*Ubn1/Ubn2* ESCs, treated and non-treated with Dox for 48h following the improved version of the original protocol (Buenrostro et al. 2013). In brief, 50000 ESCs per condition were lysed in detergent containing lysis buffer and the nuclei were incubated with TDE1 tagment DNA enzyme (Illumina, #15027865) in tagment DNA buffer (Illumina, #15027866). The tagmented DNA was purified with the MinElute PCR purification kit, eluted in 10µl EB buffer, amplified and multiplexed with Nextera DNA CD indexes (Illumina, #20015881) by PCR to sub-saturation. The libraries were purified with the MinElute PCR purification kit and eluted in

20µl EB buffer. Paired-end 75bp sequencing was performed by the sequencing service at the Functional Genomics Center Zürich (FGCZ) on a Illumina HiSeq 2500 instrument.

Data availability

Read datasets from ChIP-Seq, RNA-Seq, ATACseq, and CUT and RUN experiments were deposited in the SRA repository of the National Center for Biotechnology Information and can be accessed under the following SRA accession numbers: ChIP-Seq PRJNA574616; RNA-Seq: PRJNA574610; ATACseq and CUT&RUN: PRJNA579971

Statistics

Paired, parametric, two-tailed t-tests were performed to determine significance with a p-value threshold smaller than 0.05. Calculations were performed using Prizm Graphpad Version 7.

Supplementary Table Legends

S1 Table. Statistics of *Ubn2* mutant mouse crosses.

In a separate Excel formatted file.

S2 Table. Statistics of *Ubn1*$^{-/-}$ *Ubn2*$^{+/-}$ mouse crosses.

In a separate Excel formatted file.

S4 Table. Differentially regulated genes.

In a separate archive file containing Excel formatted files with comparisons between different genotypes and WT.

Supplementary References

Buenrostro JD, Giresi PG, Zaba LC, Chang HY, Greenleaf WJ. 2013. Transposition of native chromatin for fast and sensitive epigenomic profiling of open chromatin, DNA-binding proteins and nucleosome position. *Nature methods* **10**: 1213-1218.

Heigwer F, Kerr G, Boutros M. 2014. E-CRISP: fast CRISPR target site identification. *Nature methods* **11**: 122-123.

Kissling L, Monfort A, Swarts DC, Wutz A, Jinek M. 2019. Preparation and electroporation of Cas12a/Cpf1-guide RNA complexes for introducing large gene deletions in mouse embryonic stem cells. *Methods in enzymology* **616**: 241-263.

Labun K, Montague TG, Gagnon JA, Thyme SB, Valen E. 2016. CHOPCHOP v2: a web tool for the next generation of CRISPR genome engineering. *Nucleic Acids Res* **44**: W272-276.

Monfort A, Di Minin G, Postlmayr A, Freimann R, Arieti F, Thore S, Wutz A. 2015. Identification of Spen as a crucial factor for Xist function through forward genetic screening in haploid embryonic stem cells. *Cell Reports* **12**: 554-561.

Perez AR, Pritykin Y, Vidigal JA, Chhangawala S, Zamparo L, Leslie CS, Ventura A. 2017. GuideScan software for improved single and paired CRISPR guide RNA design. *Nature biotechnology* **35**: 347-349.
