# Supplementary material for: Ubinuclein 2 is essential for mouse development and functions in X chromosome inactivation
Source: PLoS Genet. 2025 Jun 2;21(6):e1011711. doi: 10.1371/journal.pgen.1011711 (PMC12165345; doi:10.1371/journal.pgen.1011711)
Supplement: S1 Fig — (PDF) [file pgen.1011711.s002.pdf]

A

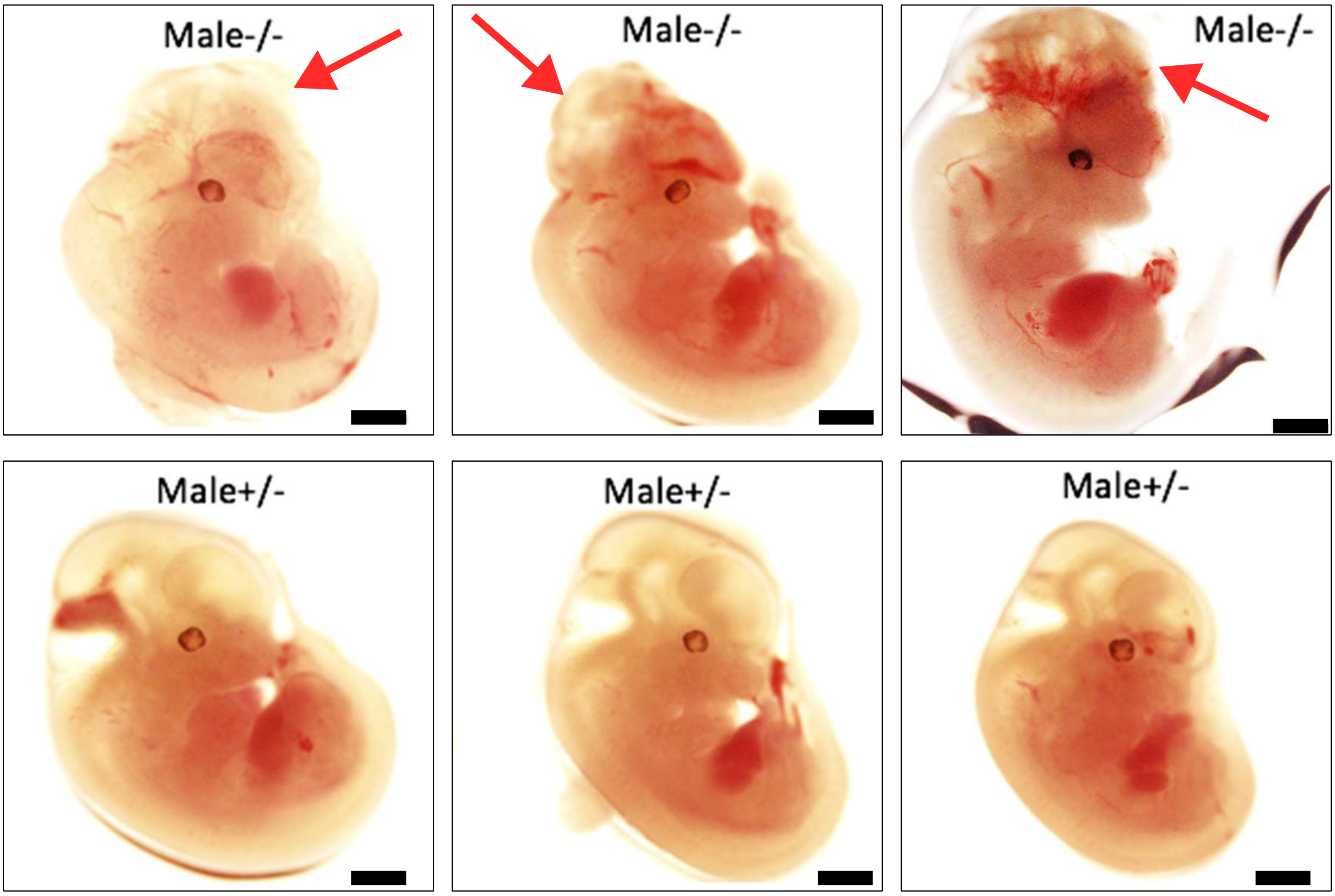

B

E12.5 embryos from an *Ubn2* -/- x *Ubn2* +/- cross

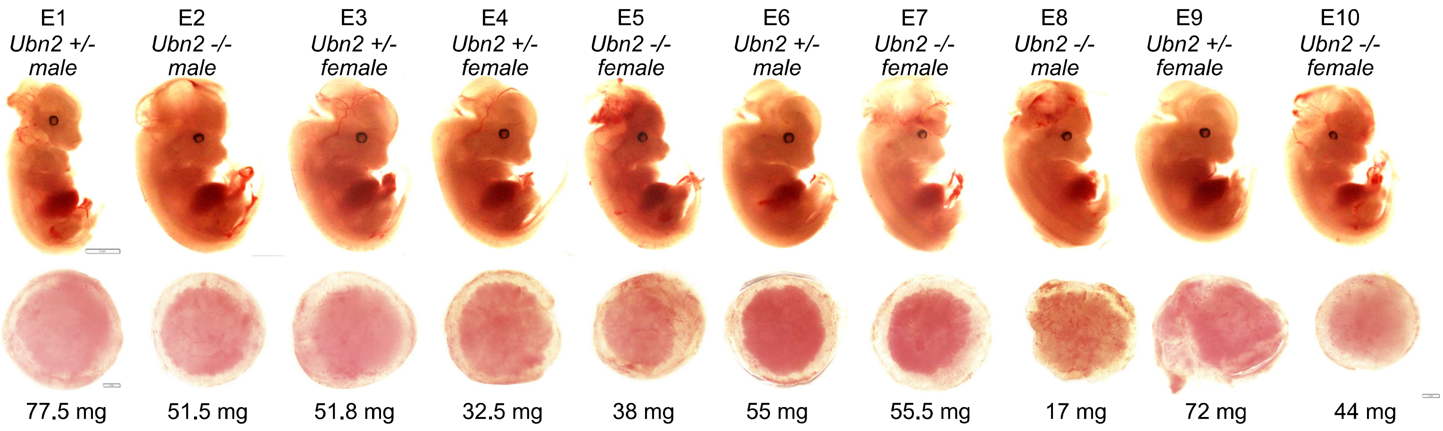

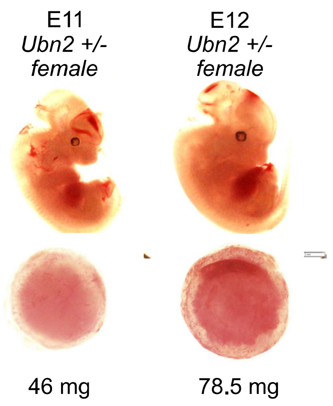

C

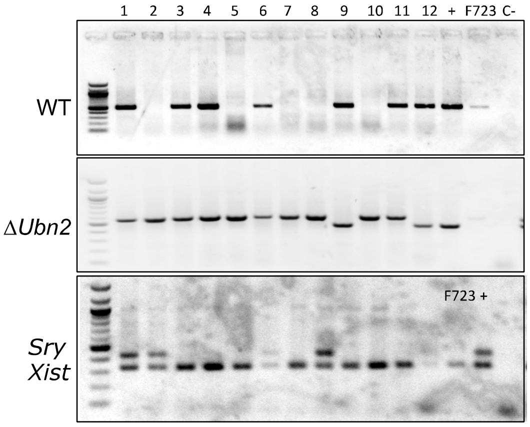

S1 Fig

S1 Fig. Homozygous and heterozygous *Ubn2* mutant male E12.5 embryos.

(**A**) Representative images of male *Ubn2*$^{-/-}$ E12.5 embryos showing exencephaly (red arrow) that might have resulted from a neural tube closure defect in the anterior head region (upper panels) and control *Ubn2*$^{+/-}$ males (below). Scale bar, 1mm. (**B**) Images or embryos and placentae dissected from one litter at E12.5. The genotype, sex of the embryo, and placental wet weight are provided. (**C**) Genotyping PCR of genomic DNA extracted from embryos shown in panel B.
