# Supplementary material for: Ubinuclein 2 is essential for mouse development and functions in X chromosome inactivation
Source: PLoS Genet. 2025 Jun 2;21(6):e1011711. doi: 10.1371/journal.pgen.1011711 (PMC12165345; doi:10.1371/journal.pgen.1011711)
Supplement: S2 Fig — (PDF) [file pgen.1011711.s003.pdf]

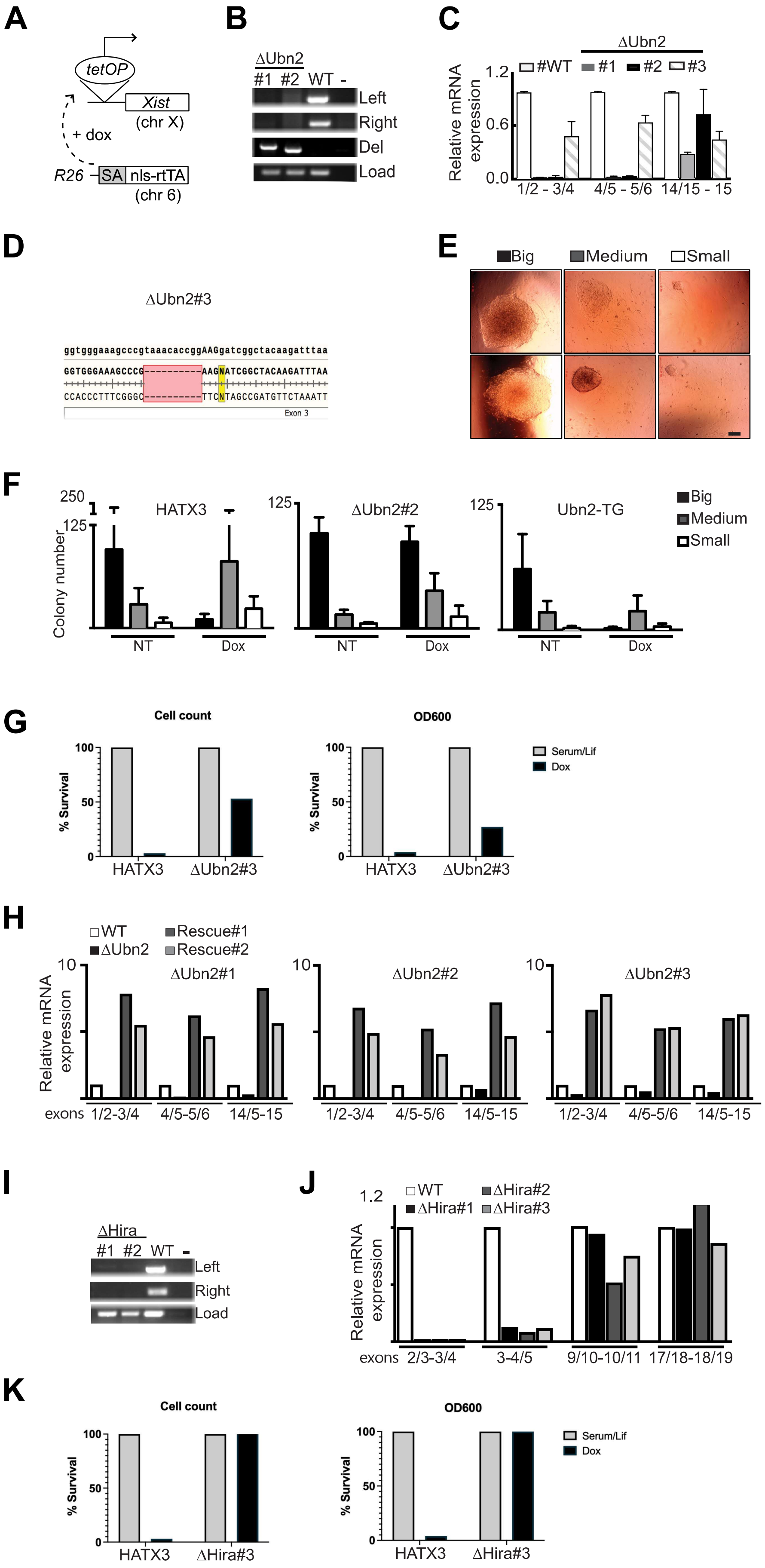
A
tetOP
Xist
(chr X)
+ dox
R26
SA
nls-rtTA
(chr 6)
B
ΔUbn2
#1 #2 WT -
Left
Right
Del
Load
C
ΔUbn2
#WT #1 #2 #3
Relative mRNA expression
1.2
0.6
0.0
1/2 - 3/4
4/5 - 5/6
14/15 - 15
D
ΔUbn2#3
ggtgggaaagcccgtaaacaccggAAGgatcggctacaagatttaa
GGTGGGAAAGCCCG
AAGNATCGGCTACAAGATTTAA
CCACCCTTTCGGGC
TTCNTAGCCGATGTTCTAAATT
Exon 3
E
Big
Medium
Small
F
250
125
HATX3
ΔUbn2#2
Ubn2-TG
Colony number
NT
Dox
Big
Medium
Small
G
Cell count
OD600
% Survival
100
50
0
HATX3
ΔUbn2#3
Serum/Lif
Dox
H
WT
ΔUbn2
Rescue#1
Rescue#2
10
ΔUbn2#1
ΔUbn2#2
ΔUbn2#3
Relative mRNA expression
exons
1/2-3/4
4/5-5/6
14/5-15
I
ΔHira
#1 #2 WT -
Left
Right
Load
J
1.2
WT
ΔHira#1
ΔHira#2
ΔHira#3
Relative mRNA expression
exons
2/3-3/4
3-4/5
9/10-10/11
17/18-18/19
K
Cell count
OD600
% Survival
100
50
0
HATX3
ΔHira#3
Serum/Lif
Dox

S2 Fig

S2 Fig. Implication of *Ubn2* and *Hira in Xist*-mediated silencing.

(A) Scheme of the Xist induction system in HATX ESCs. A doxycycline inducible promoter (tetOP) is inserted at the transcription start site of the Xist gene. The tetracycline regulated transactivator (nls-rtTA) is expressed from the ROSA26 (R26) locus. Binding of nls-rtTA to tetOP in the presence of doxycycline (dox) activates Xist expression. (B) PCR identifying deletions in ΔUbn2 clones #1 and #2. (C) RT-qPCR of Ubn2 exons as indicated for the three independent ΔUbn2 ESC clones. (D) Sanger sequencing of the Left PCR product (Fig 4A) for clone ΔUbn2#3 revealing a 10 bp deletion causing a frameshift in exon 3. (E) Representative images of big, medium, and small colonies used for quantification of the clonal survival assay. Scale bar, 1 mm. (F) Xist survival assay for control HATX3, ΔUbn2 clone #2, and ΔUbn2 clone #2 ESCs rescued with a full-length Ubn2 transgene (Ubn2-TG). For each cell line colony numbers binned in 3 size classes are shown without (NT) or with (Dox) Xist induction (n = 3). (G) Doxycycline survival assay for control HATX3 and ΔUbn2 clone #3, survival measured using cell counts determined with a hemocytometer and OD600. (H) RT-qPCR of Ubn2 exons as indicated. Three independent ΔUbn2 ESC clones along with each two independent ESC clones rescued with WT Ubn2 cDNA transgenes are shown. (I) Genomic PCR confirming the absence of wild type Hira fragment in ΔHira#1 and ΔHira#2 mutant ESCs. (J) RT-qPCR of Hira exons as indicated for three independent ΔHira mutant clones. (K) Doxycycline survival assay for control HATX3 and ΔHira clone #3, survival percentage determined by cell counting with a hemocytometer and OD600.
