# Supplementary material for: Ubinuclein 2 is essential for mouse development and functions in X chromosome inactivation
Source: PLoS Genet. 2025 Jun 2;21(6):e1011711. doi: 10.1371/journal.pgen.1011711 (PMC12165345; doi:10.1371/journal.pgen.1011711)
Supplement: S3 Fig — (PDF) [file pgen.1011711.s004.pdf]

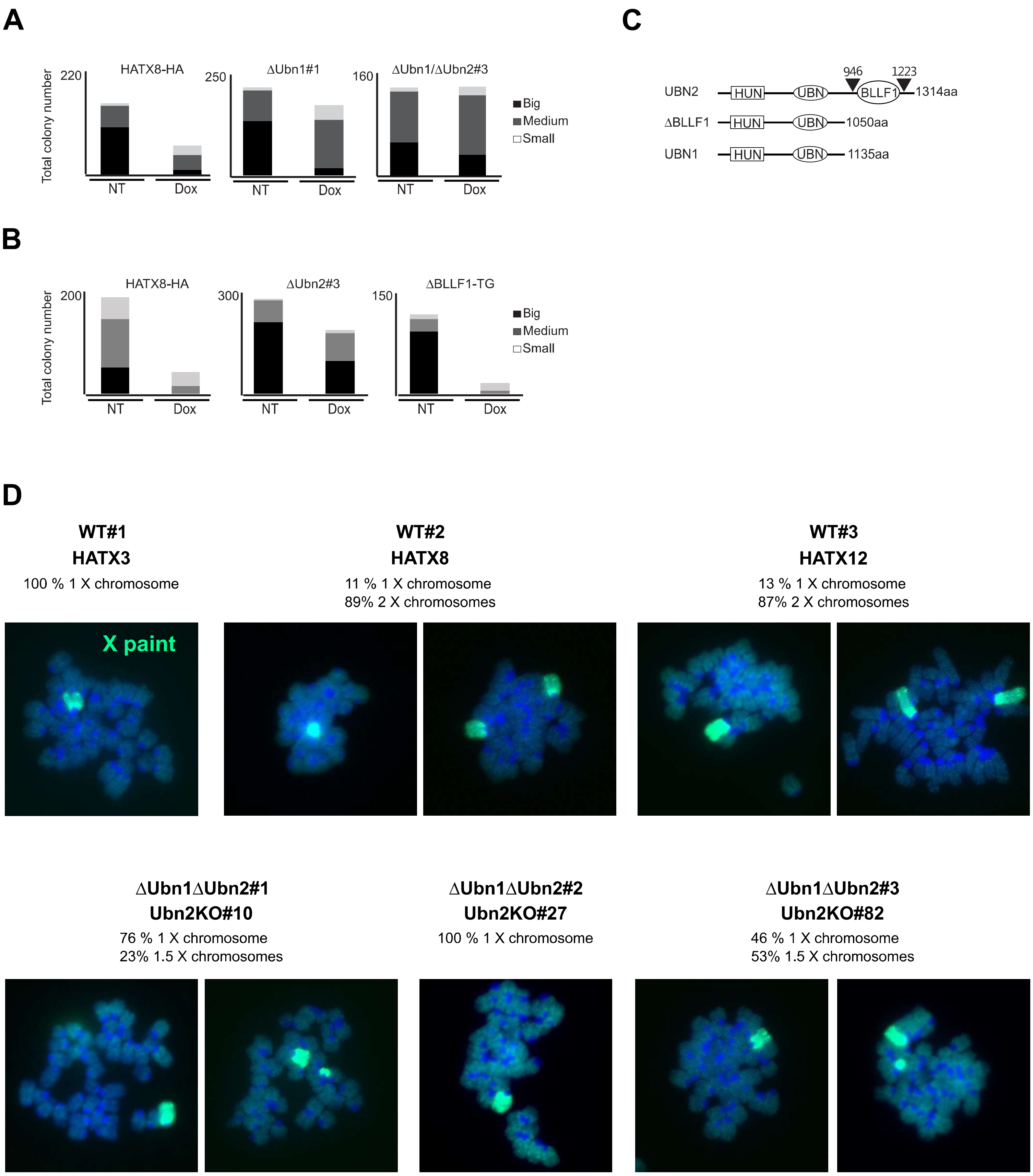
A
HATX8-HA
ΔUbn1#1
ΔUbn1/ΔUbn2#3
Total colony number
220
250
160
NT
Dox
Big
Medium
Small
B
HATX8-HA
ΔUbn2#3
ΔBLLF1-TG
200
300
150
C
UBN2
ΔBLLF1
UBN1
HUN
UBN
BLLF1
946
1223
1314aa
1050aa
1135aa
D
WT#1
HATX3
100 % 1 X chromosome
WT#2
HATX8
11 % 1 X chromosome
89% 2 X chromosomes
WT#3
HATX12
13 % 1 X chromosome
87% 2 X chromosomes
X paint
ΔUbn1ΔUbn2#1
Ubn2KO#10
76 % 1 X chromosome
23% 1.5 X chromosomes
ΔUbn1ΔUbn2#2
Ubn2KO#27
100 % 1 X chromosome
ΔUbn1ΔUbn2#3
Ubn2KO#82
46 % 1 X chromosome
53% 1.5 X chromosomes

S3 Fig

S3 Fig. *Ubn1* and *Ubn2* contribute to efficient gene repression by *Xist*.

(**A**) Representative *Xist* survival assay for control HATX8 *Ubn2*-HA, Δ*Ubn1* clone *#1, and* Δ*Ubn1/*Δ*Ubn2* clone *#3* ESCs. Colony numbers without (NT) or with *Xist* induction (Dox) are plotted. (**B**) *Xist* survival assay for control HATX8 *Ubn2*-HA ESCs, Δ*Ubn2* clone *#3* mutant ESCs, and Δ*Ubn2* clone *#3* mutant ESCs rescued with a ΔBLLF1-UBN2 cDNA transgene. (**C**) Schematic representation not drawn to scale indicating protein domains of Ubinucleins and the position of the ΔBLLF1 deletion (black triangles). (**D**) X-chromosome paint DNA FISH analysis of metaphase spreads from wild type and ΔUbn1/ΔUbn2 cells shows the number of X chromosomes. The cell line name, corresponding replicate number for WT and ΔUbn1/ΔUbn2 mutant samples as used in the figures and text, and the percentage of spreads with one and two X chromosomes is indicated above the images.
