# Supplementary material for: Ubinuclein 2 is essential for mouse development and functions in X chromosome inactivation
Source: PLoS Genet. 2025 Jun 2;21(6):e1011711. doi: 10.1371/journal.pgen.1011711 (PMC12165345; doi:10.1371/journal.pgen.1011711)
Supplement: S4 Fig — (PDF) [file pgen.1011711.s005.pdf]

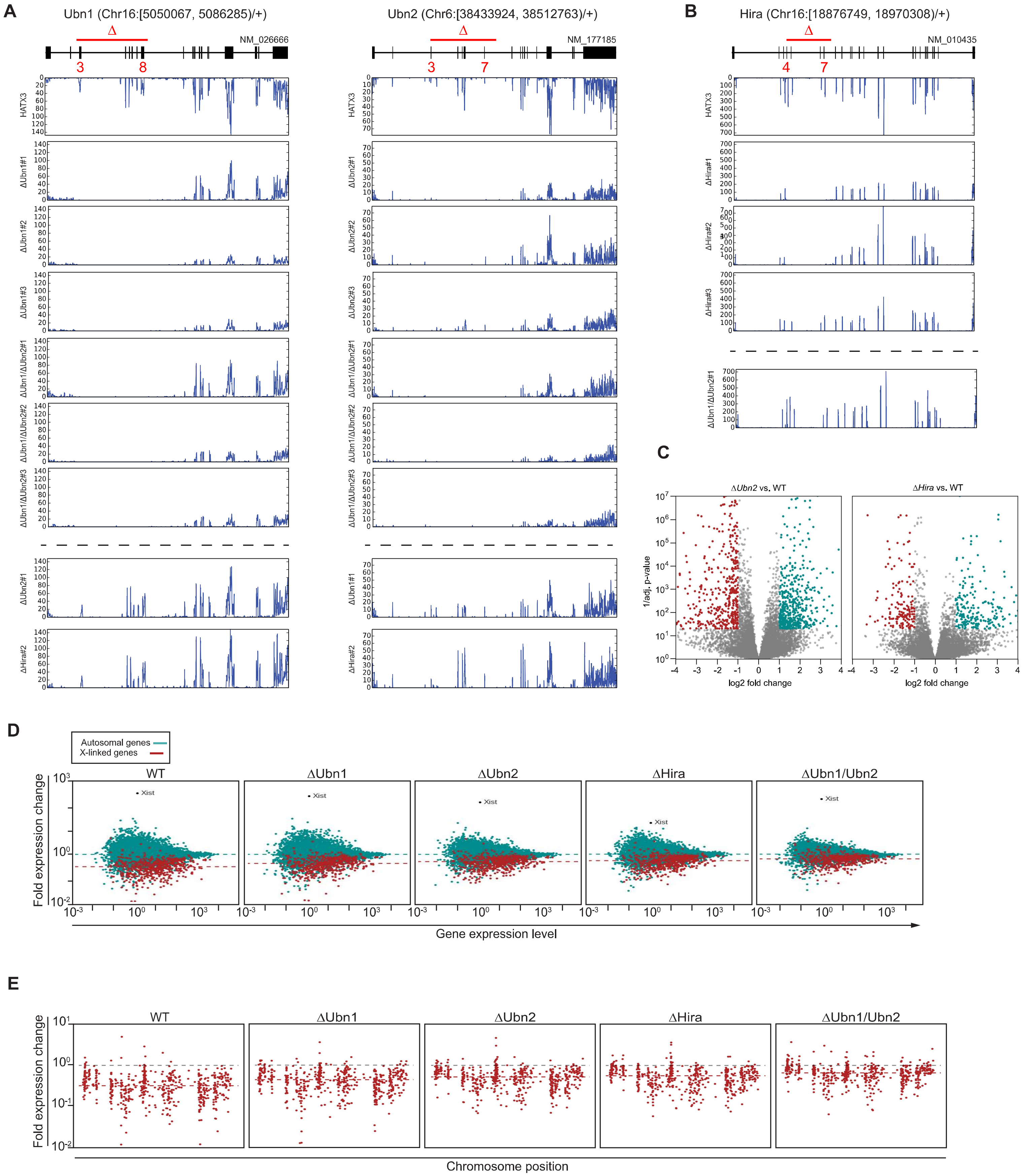
A
Ubn1 (Chr16:[5050067, 5086285)/+)
Δ
NM_026666
3
8
HATX3
ΔUbn1#1
ΔUbn1#2
ΔUbn1#3
ΔUbn1/ΔUbn2#1
ΔUbn1/ΔUbn2#2
ΔUbn1/ΔUbn2#3
ΔUbn2#1
ΔHira#2
Ubn2 (Chr6:[38433924, 38512763)/+)
NM_177185
3
7
B
Hira (Chr16:[18876749, 18970308)/+)
NM_010435
4
7
ΔHira#1
ΔHira#2
ΔHira#3
C
ΔUbn2 vs. WT
ΔHira vs. WT
1/adj. p-value
log2 fold change
D
Autosomal genes
X-linked genes
WT
ΔUbn1
ΔUbn2
ΔHira
ΔUbn1/Ubn2
Xist
Fold expression change
Gene expression level
E
Chromosome position

S4 Fig

S4 Fig. RNA expression analysis of *Ubn1*, *Ubn2* and *Hira* mutant ESCs.

(**A**) RNA-Seq read coverage profiles for the *Ubn1 and Ubn2* genes in Δ*Ubn1,* Δ*Ubn2,* and Δ*Ubn1*/Δ*Ubn2* ESC clones. (**B**) RNA-Seq read coverage profiles for the *Hira* gene in Δ*Hira#1,* Δ*Hira#2,* and Δ*Hira#3* ESC clones. Deleted regions (red lines) and outermost exons within the deletion (red numbers) are indicated above the gene models in panels A and B. (**C**) Volcano plots depict the fold expression change for the indicated genotypes relative to wild-type. Genes that are at least 2-fold significantly (adjusted p-value < 0.05 calculated using DESeq2) up-regulated (green) and down-regulated (red) are marked. (**D**) Fold change expression of autosomal (green) and X-linked genes (red) after 48h of Dox treatment for ESCs of genotypes indicated versus gene expression level in untreated conditions. *Xist* is indicated. Dotted lines indicate the median fold change for autosomal (green) and X-linked genes (red). (**E**) Differential X-linked expression fold change after 48h of Dox treatment for ESCs of genotypes indicated over the position of the genes on the X chromosome. Red dotted lines indicate the median fold change for three independent replicates, black dotted lines at fold change of 1.
