# Supplementary material for: Ubinuclein 2 is essential for mouse development and functions in X chromosome inactivation
Source: PLoS Genet. 2025 Jun 2;21(6):e1011711. doi: 10.1371/journal.pgen.1011711 (PMC12165345; doi:10.1371/journal.pgen.1011711)
Supplement: S5 Fig — (PDF) [file pgen.1011711.s006.pdf]

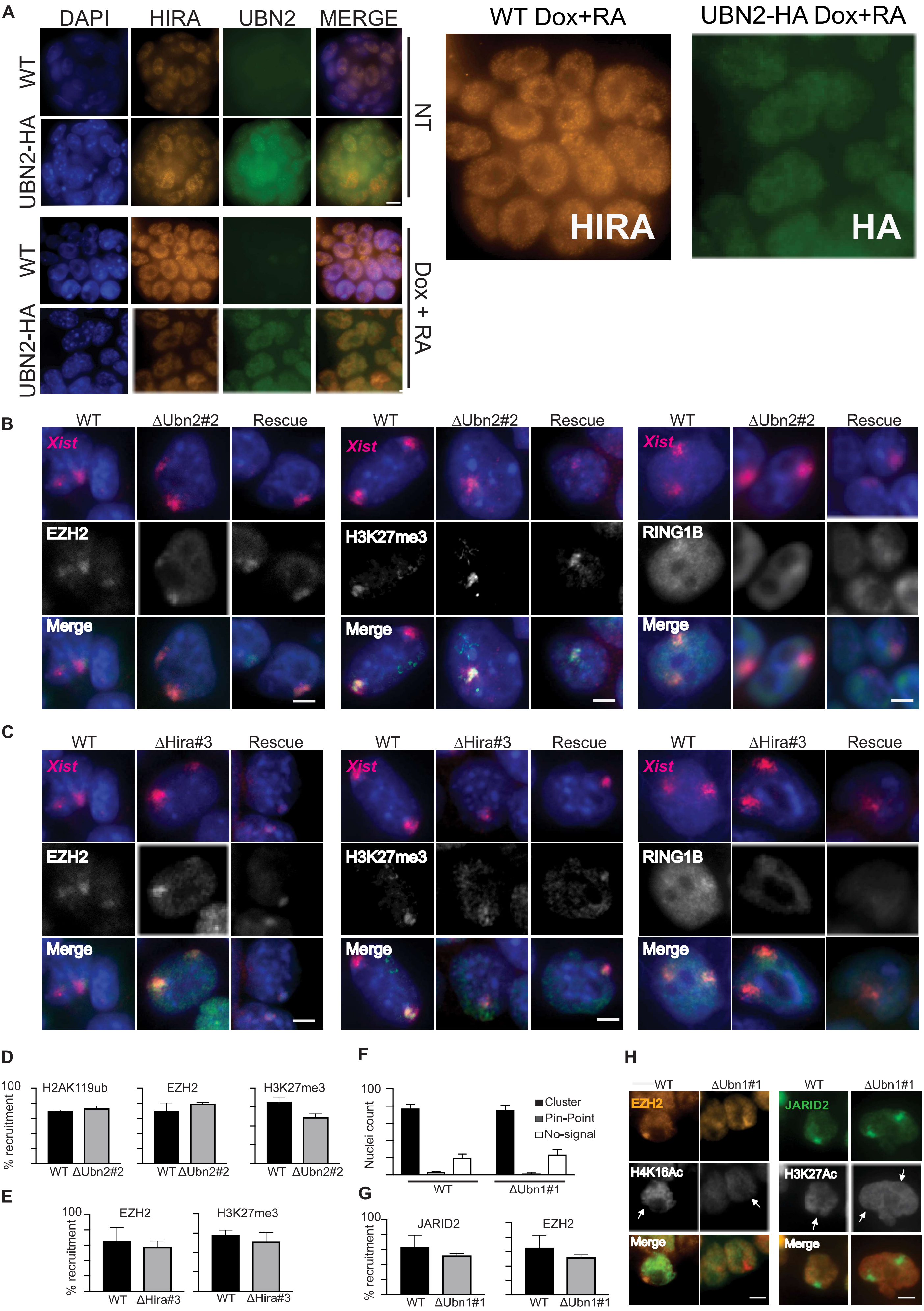

A
DAPI
HIRA
UBN2
MERGE
WT
UBN2-HA
NT
Dox + RA
WT Dox+RA
UBN2-HA Dox+RA
HIRA
HA
B
WT
ΔUbn2#2
Rescue
Xist
EZH2
H3K27me3
RING1B
Merge
C
ΔHira#3
D
100
% recruitment
H2AK119ub
EZH2
H3K27me3
WT ΔUbn2#2
E
WT ΔHira#3
F
Nuclei count
Cluster
Pin-Point
No-signal
WT
ΔUbn1#1
G
JARID2
H
H4K16Ac
H3K27Ac

S5 Fig

S5 Fig. *Hira* and Ubinucleins are not required for the formation of a repressive compartment. (**A**) Immunofluorescence staining in WT and *Ubn2*-HA ESCs showing punctate nuclear staining for HIRA and UBN2-HA after 48h of *Xist* induction (Dox) and retinoic acid treatment (RA) or uninduced (NT). Enlarged views of HIRA and UBN2-HA after Xist induction are shown on the right. Scale bar, 10 µm. (**B,C**) Combined *Xist* RNA FISH and immunofluorescence staining showing focal EZH2, H3K27me3 and RING1B staining over the *Xist* cluster in Δ*Ubn2#2* (**B**) and Δ*Hira#3* (**C**) mutant ESCs after 24h of *Xist* induction. (**D**) Quantification of H2AK119ub, EZH2 and H3K27me3 recruitment in Δ*Ubn2#2* mutant ESCs after 24h of *Xist* induction. (**E**) Quantification of EZH2 and H3K27me3 recruitment in Δ*Hira#3* mutant ESCs after 24h of *Xist* induction. (**F**) Statistical analysis of *Xist* clusters from *Xist* RNA FISH experiments for Δ*Ubn1#1* ESCs. (**G**) Quantification of JARID2 and EZH2 recruitment in Δ*Ubn1#1* mutant ESCs after 24h of *Xist* induction. (**H**) Double immunofluorescence staining of H4K16ac and EZH2 as well as JARID2 and H3K27ac in Δ*Ubn1#1* mutant ESCs after 24h of *Xist* induction showing EZH2 foci are depleted in H4K16ac, and JARID2 foci are depleted in H3K27ac. Depleted regions overlapping with EZH2 and JARID2 foci are indicated by white arrows. Scale bars in A, B, C, H are 5 µm.
