# Supplementary material for: Ubinuclein 2 is essential for mouse development and functions in X chromosome inactivation
Source: PLoS Genet. 2025 Jun 2;21(6):e1011711. doi: 10.1371/journal.pgen.1011711 (PMC12165345; doi:10.1371/journal.pgen.1011711)
Supplement: S6 Fig — (PDF) [file pgen.1011711.s007.pdf]

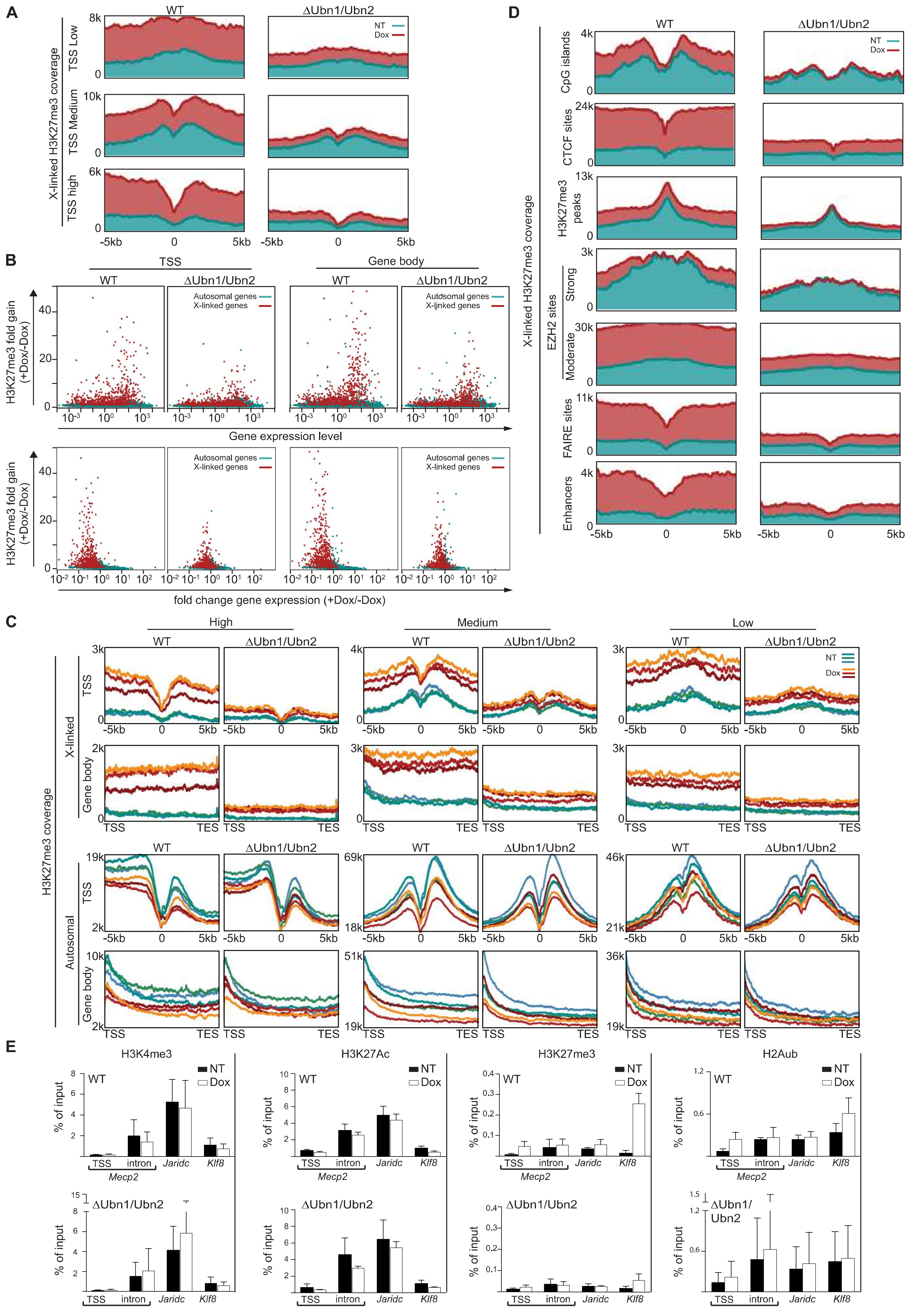
A
WT
ΔUbn1/Ubn2
NT
Dox
X-linked H3K27me3 coverage
TSS Low
TSS Medium
TSS high
8k
10k
6k
0
-5kb
0
5kb
B
TSS
Gene body
Autosomal genes
X-linked genes
H3K27me3 fold gain (+Dox/-Dox)
Gene expression level
fold change gene expression (+Dox/-Dox)
C
High
Medium
Low
H3K27me3 coverage
X-linked
Autosomal
TSS
Gene body
TES
D
X-linked H3K27me3 coverage
CpG islands
CTCF sites
H3K27me3 peaks
EZH2 sites
Strong
Moderate
FAIRE sites
Enhancers
4k
24k
13k
3k
30k
11k
E
H3K4me3
H3K27Ac
H3K27me3
H2Aub
% of input
TSS
intron
Mecp2
Jaridc
Klf8
ΔUbn1/Ubn2

S6 Fig

S6 Fig. Establishment of H3K27me3 over X-linked genes requires Ubinucleins.

(**A**) Profiles of H3K27me3 coverage over X-linked promoters (TSS) for WT and ΔUbn1/ΔUbn2 ESCs either without (NT) or after 48h of *Xist* induction (Dox). Genes are grouped by expression level as high, medium and low. (**B**) Fold change of H3K27me3 over X-linked gene TSS (left) or gene body (right) after 48h of Dox treatment is plotted over the gene expression level in WT for WT and ΔUbn1/ΔUbn2 ESCs (upper panels). Fold change of H3K27me3 over X-linked gene TSS (left) or gene body (right) after 48h of Dox treatment is plotted over the fold change of gene expression (+Dox/-Dox) for WT and ΔUbn1/ΔUbn2 ESCs (lower panels). Values for autosomal (blue), and X-linked genes (red) were calculated using three independent replicates. (**C**) Profiles of H3K27me3 coverage over X-linked and autosomal promoters (TSS) and gene bodies for three independent WT and ΔUbn1/ΔUbn2 ESC clones either without (NT) or after 48h of *Xist* induction (DOX). Genes are grouped by expression level as high, medium and low. (**D**) Cumulative H3K27me3 coverage over X-linked CpG islands, CTCF sites, H3K27me3 peaks, EZH2 strong and moderate sites, FAIRE sites, and enhancers for WT and ΔUbn1/ΔUbn2 ESCs either without (NT) or after 48h of *Xist* induction (Dox). (**E**) ChIP with antibodies against H3K4me3, H3K27ac, H3K27me3 and H2Aub over three X-linked gene promoters, *Mecp2*, *Jaridc* and *Klf8*, before (NT) and after (Dox) *Xist* induction for 48h in WT and Δ*Ubn1/*Δ*Ubn2#1* ESCs (n = 3).
