# Supplementary material for: Ubinuclein 2 is essential for mouse development and functions in X chromosome inactivation
Source: PLoS Genet. 2025 Jun 2;21(6):e1011711. doi: 10.1371/journal.pgen.1011711 (PMC12165345; doi:10.1371/journal.pgen.1011711)
Supplement: S7 Fig — (PDF) [file pgen.1011711.s008.pdf]

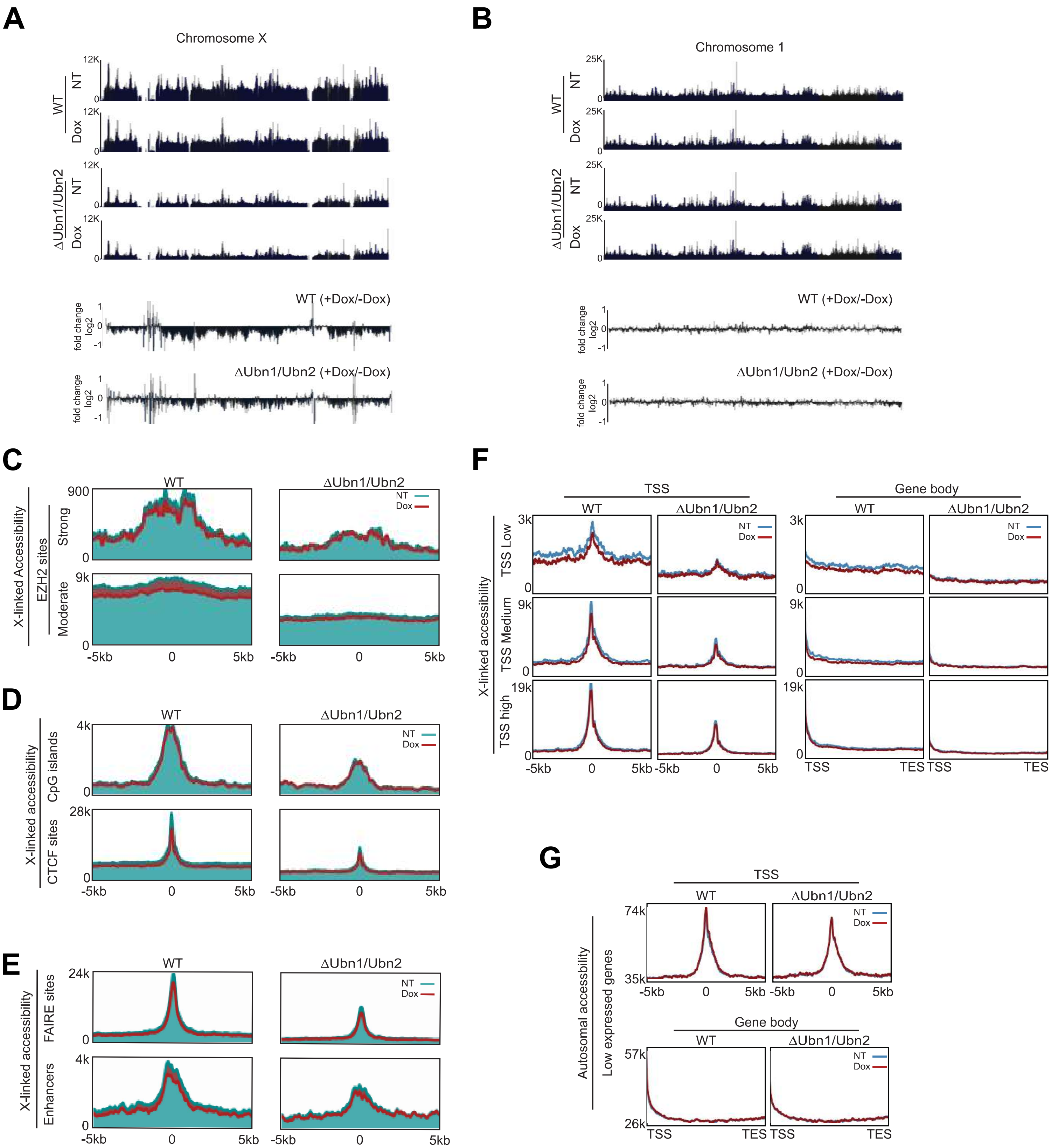
A
Chromosome X
WT
NT
Dox
ΔUbn1/Ubn2
12K
0
WT (+Dox/-Dox)
ΔUbn1/Ubn2 (+Dox/-Dox)
fold change log2
B
Chromosome 1
25K
C
X-linked Accessibility
EZH2 sites
Strong
Moderate
900
9k
-5kb
0
5kb
D
X-linked accessibility
CpG islands
CTCF sites
4k
28k
E
FAIRE sites
Enhancers
24k
F
TSS
Gene body
TSS Low
TSS Medium
TSS high
3k
19k
TES
G
Autosomal accessibility
Low expressed genes
74k
35k
57k
26k

S7 Fig

S7 Fig. Effect of loss of Ubinucleins on chromatin accessibility.

(**A**) Chromatin accessibility from ATACseq over the X chromosome and (**B**) chromosome 1 in WT and ΔUbn1/ΔUbn2 ESCs either without (NT) or after 48h of *Xist* induction (Dox, n=1). The log2 fold change of accessibility after *Xist* induction is plotted below (+Dox/-Dox) for both genotypes. (**C**) ATACseq coverage profiles over X-linked moderate and strong EZH2 sites, (**D**) CpG islands and CTCF sites, and (**E**) FAIRE sites and enhancers in WT and ΔUbn1/ΔUbn2 ESCs either without (NT) or after 48h of *Xist* induction (Dox, n=1). (**F**) Profiles of ATACseq coverage in WT and ΔUbn1/ΔUbn2 ESC either without (NT) or after 48h of *Xist* induction (Dox) over X-linked TSS and gene bodies of high, medium and low expressed genes (n=1), and (**G**) over autosomal TSS and gene bodies in low expressed genes (n=1).
