# Supplementary material for: Ubinuclein 2 is essential for mouse development and functions in X chromosome inactivation
Source: PLoS Genet. 2025 Jun 2;21(6):e1011711. doi: 10.1371/journal.pgen.1011711 (PMC12165345; doi:10.1371/journal.pgen.1011711)
Supplement: S3 Table — (PDF) [file pgen.1011711.s011.pdf]

S3 Table. SILAC mass spectrometry of HA-Ubn2 interactors. HA-immunoprecipitation from heavy labelled control ESCs and light labelled *Ubn2*-HA ESCs are compared. Asterisks indicate control proteins with no enrichment.

Uniprot Accession Number	Gene name	Length (aa)	Unique peptides		Total peptides
			Light	Heavy	
Q61666	*Hira*	1015	10	0	11
Q4G0F8	*Ubn1*	1135	5	0	5
O88480	*Cabin1*	2182	11	0	11
Q9Y6J0	*Cabin1*	2220	8	0	8
P80316	*Cct5*	541	7	0	8
P80317	*Cct6a*	531	8	0	9
P11983	*Tcp1*	556	7	0	8
P42932	*Cct8*	548	7	0	7
P11276*	*Finc*	2477	34	53	87
P14733*	*Lmnb1*	588	35	28	64
