# Supplementary material for: Ubinuclein 2 is essential for mouse development and functions in X chromosome inactivation
Source: PLoS Genet. 2025 Jun 2;21(6):e1011711. doi: 10.1371/journal.pgen.1011711 (PMC12165345; doi:10.1371/journal.pgen.1011711)
Supplement: S5 Table — (PDF) [file pgen.1011711.s013.pdf]

S5 Table. Sequences of primers and gRNAs used in this study.

**Primer sequences**		
**CRISPR gRNA sequences**		
**Gene**	**System**	**Sequence**
Ubn2 exon 3 Fw	pX458	*CACCG* AAAGCCCGTAAACACCGGA
Ubn2 exon 3 Rv	pX458	*AAAC* TCCGGTGT TTACGGGCT TT *C*
Ubn2 intron 7 Fw	pX458	*CACCG* ACCCGGGATTCGCATCAGCT
Ubn2 intron 7 Rv	pX458	*AAAC* AGCTGATGCGAATC CCG GGT *C*
Hira exon 4 Fw	pX458	*CACCG* CGCACACAGTTCACACATGC
Hira exon 4 Rv	pX458	*AAAC* GCATGTGTGAACTG TGTGCG *C*
Hira intron 7 Fw	pX458	*CACCG* TTGACCATGAACACCTCAT
Hira intron 7 Rv	pX458	*AAAC* ATGAGGTGTTCATGGTCAA *C*
Ubn2 C-term Fw	pX458	*CACCG* CCCTTGCTGGAAAGTCACTG
Ubn2 C-term Rv	pX458	*AAAC* CAGTGACTTTCCAGCAAGGG *C*
Ubn2 exon3	Cas12aRNP	TGGAAACAGTACAGGTACTG
Ubn2 intron 7	Cas12aRNP	CAGGCTGCTGAGAATATAGTT
Ubn1 intron 2	Cas12aRNP	GTTTGACCTGGCCCTTCTTT
Ubn1 intron 8	Cas12aRNP	CCTCAGTTGCCTGCTTGTCA
Ubn2 exon 3	Cas9RNP	AAAGCCCGTAAACACCGGA
Ubn2 intron 7	Cas9RNP	ACCCGGGATTCGCATCAGCT
Ubn2 BLLF right	Cas9RNP	AGGCCACCCAGCATCCCAAA
Ubn2 BLLF left	Cas9RNP	AAAGGGAGGCGATACATCAG
**Primer sequences used to validate gene editing**		
**Primer name**		**Sequence (5' to 3')**
Ubn2 intron 2 Fw		GCAAGGTATGGGGAGATCCAG
Ubn2 intron 3 Rv		CACCATATGGCCCACAGTCG
Ubn2 intron 6 Fw		GAGTCTGTGCTGTTAAAGCCACTG
Ubn2 intron 7 Rv		ACATGTATGCAGATGTGGGTGAATGC
Ubn2 intron 16Fw		TGCTTTAGAACCCAGCATTG
Ubn2 exon 17 Rv		CAAGCATGTAAACATGGACAG
Ubn1 intron 2 Fw		ACAACAAACCCCAAGTTTGGACTG
Ubn1 intron 3 Rv		GGTCCATGTGAACACTGGTAGC
Ubn1 exon 8 Fw		ACTCTCTGACGGATTTGGAC
Ubn1 intron 8 Rv		CCATGCTAAAGACTGCTTGC
Ubn1 del Fw		ACTGACTGGGGGATTTCTTC
Ubn1 del Rv		TGATGTTCTGCACTGTCCTG
Hira intron 3 Fw		GGCATGCCTATTGGATTTTC
Hira intron 4 Rv		TTGATAAAGACCAGCAAAGAAGC
Hira exon 7 Fw		CTCTGAGAGGTCATTCTGGC
Hira intron 7 Rv		GGGAAGGAGGTTACCAACG
**Primer sequences used for gene expression analysis**		
**Primer name**		**Sequence (5' to 3')**
Ubn2 1/2 Fw		TGTGCGGAGAACAACGGAAG
Ubn2 3/4 Rv		GCAGGGACTAATTCGTCATAAGCC
Ubn2 4/5 Fw		GCACAAGCCACCCAAGGTTC

Ubn2 5/6 Rv		GAGCCACAACTCCCAGTTGC
Ubn2 14/15 Fw		TAACAGCCAGTGTGCAGTCC
Ubn2 e15 Rv		GTCCGTTCCAAAGCCAAGTA
Ubn1 5/6 Fw		GGCGTATGATGAACTTGTTCCTGC
Ubn1 6/7 Rv		CCTTCAACTTCCGCTTCTTCGGAG
Ubn1 8/9 Fw		TGTTAAGGAGCTGGCTCAGGC
Ubn1 9/10 Rv		CGAGTTTGCACCTCTATGTCCAGC
Ubn1 11/12 Fw		GGCAAAGGTTGCTAAGATGCTGG
Ubn1 12/13 Rv		CCTGGCACAGAAGCTCCCTG
Hira 2/3 Fw		CAACTGGAGGACAAGGGCAG
Hira 3/4 Rv		CGCACACAGTTCACACATGCT
Hira e3 Fw		GTCCTCCAGGAGGATGACGAG
Hira 4/5 Rv		CAATGTACGTAGCCCGCTTCC
Hira 9/10 Fw		CACTCTCTGTCTGGCTCACATG
Hira 10/11 Rv		CCAACCCATTCAGAGTCCAGG
Hira 17/18 Fw		CCTCCAGGTGAGCTCTGACC
Hira 17/18 Fw_2		CGTTCACCCTCCAGGTGAGC
Hira 18/19 Rv		CATACCACATCACAGCTGCCG
Rrm2 Fw		CCGAGCTGGAAAGTAAAGCG
Rrm2 Rv		ATGGGAAAGACAACGAAGCG
Actb Fw		CTAAGGCCAACCGTGAAAAG
Actb Rv		GGGGTGTTGAAGGTCTCAAA
Eif4a2 Fw		ACACCATCGGGGTCCATTCC
Eif4a2 Rv		CCTGTCTTTTCAGTCGGGCG
Sdha Fw		TTCCGTGTGGGGAGTGTATTGC
Sdha Rv		AGGTCTGTGTTCCAAACCATTCC
**Primer sequences used for mice genotyping**		
**Primer name**		**Sequence (5' to 3')**
Ubn2 intron 2 Fw		GCAAGGTATGGGGAGATCCAG
Ubn2 intron 3 Rv		CACCATATGGCCCACAGTCG
Ubn2 intron 6 Fw		GAGTCTGTGCTGTTAAAGCCACTG
Ubn2 intron 7 Rv		ACATGTATGCAGATGTGGGTGAATGC
Hira intron 5 Fw		CCATTTAGCTTTGCTTGCAGG
Hira exon 7 Rv		ACCTCATCAAAAGGCTTGGTG
Sry Fw		AAGCTTTGCTGGTTTTTGGAGTAC
Sry Rv		CTCATCGGAGGGCTAAAGTGTC
**Primer sequences used ChIP-qPCR**		
**Primer name**		**Sequence (5' to 3')**
Wnt5a Fw		AATGAAAGGACAAAATTTGGCG
Wnt5a Rv		GGACCACAGAACAATCAGGCAC
Gapdh Fw		GGGCACTCCAAGATCAACACTA
Gapdh Rv		AGGCTCAAGGGCTTTTAAGG
Mecp2 Fw_1		AAGGTGGCTCCAGGGAGAT
Mecp2 Rv_1		GTGGCTTTCTCCACTCGTCT
Mecp2 Fw_2		GCTCTTGGCGCCTATTAGAGC
Mecp2 Rv_2		CTCCTCAACAGGCAACTTTGCTAC

JaridC Fw		CGCGACTGGGACTTAACTGTAG
JaridC Rv		GTCCTGTTCCGTTTCTTCCACAC
Klf8 Fw		TCAACTTTGCGTTCAAGTCGTGATC
Klf8 Rv		ATTCTCCACCCACCTCAGAACTC
