# Supplementary material for: Ubinuclein 2 is essential for mouse development and functions in X chromosome inactivation
Source: PLoS Genet. 2025 Jun 2;21(6):e1011711. doi: 10.1371/journal.pgen.1011711 (PMC12165345; doi:10.1371/journal.pgen.1011711)
Supplement: S6 Table — (PDF) [file pgen.1011711.s014.pdf]

S6 Table. Reagents, instruments, and materials used in this study.

	Supplier	Item identifier or order number
**Antibodies**		
Mouse monoclonal HIRA (WC119)	Millipore	Cat#04-1488; RRID:AB_1977097
Mouse monoclonal alpha TUBULIN (1F4E3)	GenScript	Cat #A01410; RRID:AB_1968943
Mouse monoclonal TUBULIN (AA2)	Sigma	Cat #T8328; RRID:AB_1844090
Goat polyclonal SOX2	R&D	Cat #AF2018; RRID:AB_355110
Mouse monoclonal beta-ACTIN-Peroxidase (AC-15)	Sigma	Cat #A3854; RRID:AB_262011
Rabbit monoclonal Lamin B1 (D4Q4Z)	Cell Signaling	Cat#12586
Rat monoclonal HA-Peroxidase (3F10)	Roche	Cat #12013819001; RRID:AB_390917
Donkey anti mouse IgG Peroxidase (H+L)	Jackson Lab	Cat #715-035-150; RRID:AB_2340770
Bovine anti goat IgG Peroxidase (H+L)	Jackson Lab	Cat #805-035-180; RRID:AB_2340874
Rabbit polyclonal UBN2	ABclonal	Cat #A10516; RRID:AB_2772789
Rabbit monoclonal JARID2 (D6M9X)	Cell signaling	Cat #13594; RRID:AB_2798269
Mouse monoclonal EZH2 (AC22)	Cell signaling	Cat #3147; RRID:AB_10694383
Rabbit polyclonal H3K27me3 (39155)	Active Motif	Cat #39155; RRID:AB_2561020
Mouse H3K27me3	Active Motif	Cat #61017; RRID:AB_2614987
Rabbit monoclonal RING1b (D22F2)	Cell signaling	Cat #5694; RRID:AB_10705604
Rabbit monoclonal H2AK119ub (D27C4)	Cell signaling	Cat #8240; RRID:AB_10891618
Mouse monoclonal POLII (8WG16)	Covance	Cat #MMS-126(8WG16); RRID:AB_10013665
Rabbit polyclonal H4ac	Millipore	Cat #06-866; RRID:AB_310270
Rabbit polyclonal H4K16ac	Millipore	Cat #07-329; RRID:AB_310525
Mouse monoclonal H3K27ac (0309)	Active Motif	Cat #39685; RRID:AB_2793305
H3K27ac (for CUT&RUN)	Cell Signaling Technology	Cat #8173
H3K4me3 (for CUT&RUN)	Cell Signaling Technology	Cat #9751
Alexa Fluor 488 donkey anti mouse IgG (H+L)	Jackson Lab	Cat #715-545-150; RRID:AB_2340846
Alexa Fluor 488 donkey anti rabbit IgG 488 (H+L)	Jackson Lab	Cat #711-545-152; RRID:AB_2313584
Cy3 donkey anti mouse IgG (H+L)	Jackson Lab	Cat #715-165-150; RRID:AB_2340813
Mouse monoclonal H3K4me3 (0304)	Active Motif	Cat #61379; RRID:AB_2793611
Rabbit polyclonal H3K27ac	Active Motif	Cat #39135; RRID:AB_2614979
Rabbit monoclonal H3K27me3 (C36B11)	Cell signaling	Cat #9733; RRID:AB_2616029
Rabbit monoclonal H2AK119ub (D27C4)	Cell signaling	Cat #8240; RRID:AB_10891618
Normal mouse Ig G	Santa Cruz	Cat #sc-2025; RRID:AB_737182
Normal rabbit Ig G	Cell signaling	#2729S; RRID:AB_1031062
**Reagents**		
Lipofectamine 2000	Invitrogen	Cat #11668-030
Proteinase K	AppliChem	Cat #A3830,0100
Doxycycline	Sigma	Cat #D9891
AsCas12a	Jinek lab	Kissling et al. 2018
SpCas9	Jinek lab	Kissling et al. 2018
DNAse I	Qiagen	Cat #79254
Phusion High fidelity DNA Polymerase	NEB	Cat #M0530S
AEBSF	Sigma	Cat #A8456
PMSF	Sigma	Cat #P7626
Trypsin inhibitor	Roche	Cat #10109886001

PepA	Sigma	Cat #P-4265
Antipain	Sigma	Cat #A-6191
Aprotinin	Roche	Cat #1-583794
Benzamidine HCL	Sigma	Cat #B-6506
Leupeptin	Roche	Cat #1-034626
NaF	Sigma	Cat #S7920
Na3VO4	Sigma	Cat #S6508
NaPPi	Sigma	Cat #221368
Benzonase nuclease	Sigma	Cat #E1014
anti-HA affinity matrix	Roche	Cat #11815016001
RiboLock RNAse inhibitor	Thermo Scientific	Cat #EO0381
Cot-1 DNA	Invitrogen	Cat #18440-016
salmon sperm DNA	Invitrogen	Cat #15632-011
tRNA	Invitrogen	Cat # AM7119
Hybrisol VII	MP Biomedicals	
Complete Mini	Roche	Cat #11836153001
Micrococcal nuclease	Cell signalling	Cat #10011S
Dynabeads protein G	ThermoFisher	Cat #10004D
TDE1 tagment DNA enzyme	Illumina	Cat #15027865
Tagment DNA buffer	Illumina	Cat #15027866
RNeasy Mini kit	Qiagen	Cat #74104
PrimeScript RT Master Mix	Takara	Cat #RR036A
Superscript III	Invitrogen	Cat #18080-051
Prime-it II	Stratagen	Cat #300385
MinElute PCR purification kit	Qiagen	Cat #28004
TruSeq Stranded mRNA kit	Illumina	Cat #20020595
NEBNext® Ultra™ II DNA Library Prep Kit	NEB	Cat #E7103
Alt-R® CRISPR-Cas9 tracrRNA, 20 nmol	IDT	Cat #1072533
CRISPR-Cas9 tracrRNA	Synthego, Microsynth	Gift
CRISPR-Cas9 crRNA	Synthego, Microsynth, IDT	sequences in Suppl. Table 5
CRISPR-Cas12a sgRNA	IDT	sequences in Suppl. Table 5
Nextera DNA CD indexes	Illumina	Cat #20015881
CUT&RUN Assay Kit	Cell Signaling Technology	Cat #86652
Spin Columns	Cell Signaling Technology	Cat #14209
**Recombinant DNA**		
pSpCas9(BB)-2A-GFP	Addgene	Cat #48138 ; RRID:Addgene_48138 *Ran Fa et al. 2013*
PB-EF1α-MCS-IRES-RFP	SBI	Cat # PB531A-2
Ubn2 cDNA	Dharmacon	Cat #MMM1013-202770327
Cy3 dCTP	GE Healthcare Amersham	
**Software and Algorithms**		

Zen 2 core imaging software	Zeiss	
MaxQuant 1.6.2.10	Miltenyi Biotec	
Scaffold4 proteomis software	Proteome Software	
**Instruments**		
HiSeq 2500	Illumina	
NextSeq 2000	Illumina	
MySeq	Illumina	
Novaseq 6000	Illumina	
Orbitrap Q-Exactive Mass Spectrometer	Thermo Scientific	
LightCycler 480 Instrument II	Roche	
