# Supplementary figures and images for: Ubinuclein 2 is essential for mouse development and functions in X chromosome inactivation
Source: PLoS Genet. 2025 Jun 2;21(6):e1011711. doi: 10.1371/journal.pgen.1011711 (PMC12165345; doi:10.1371/journal.pgen.1011711)

Panel B

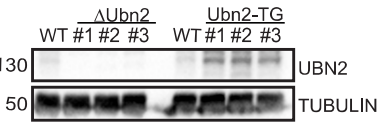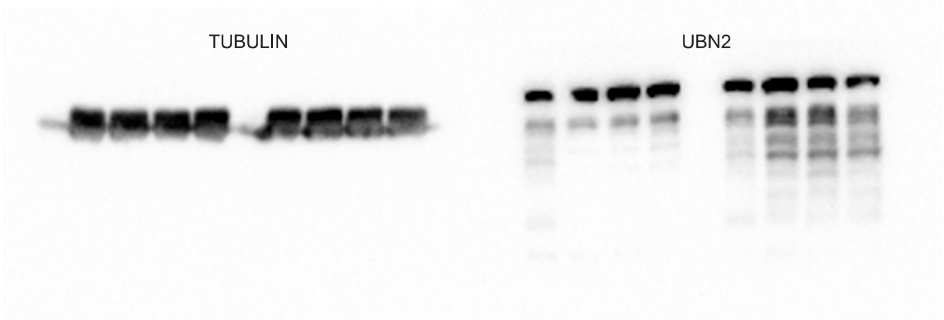

Panel E

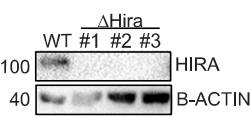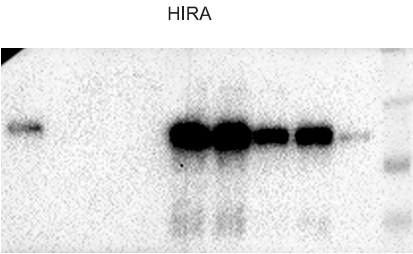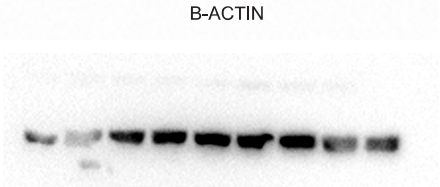

Panel G

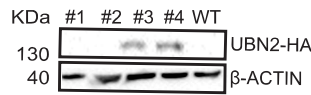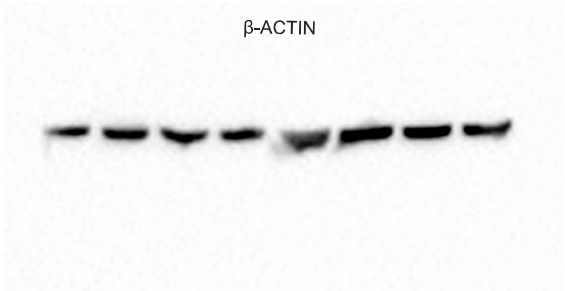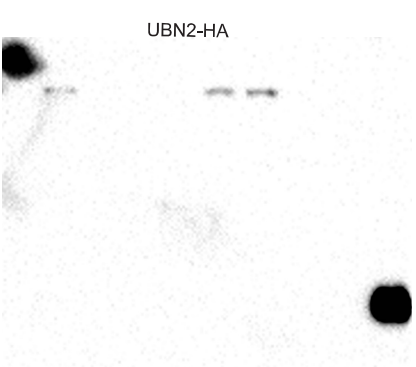

Panel H

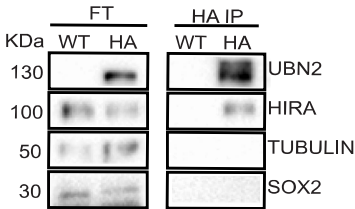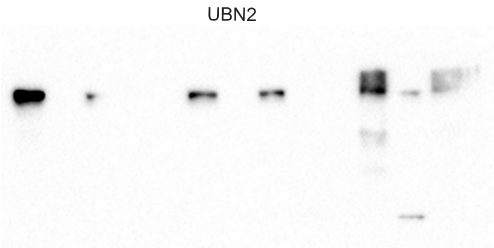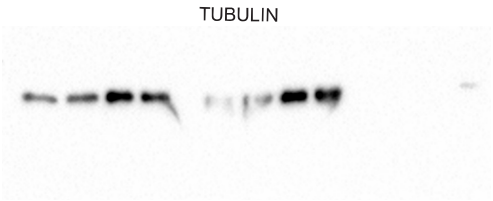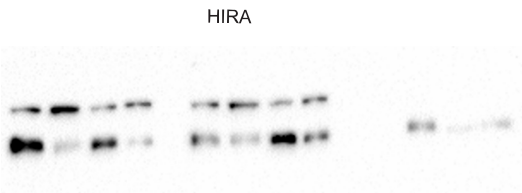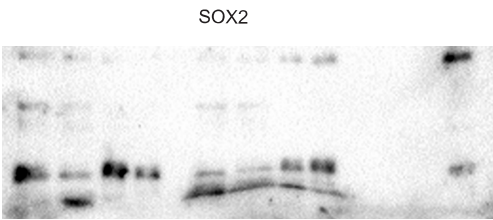

Panel L

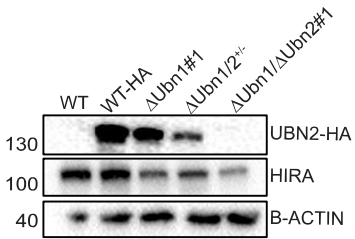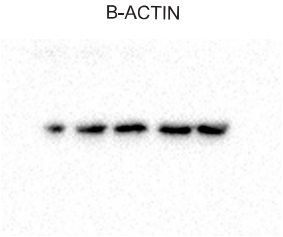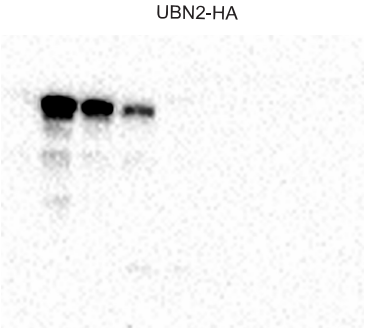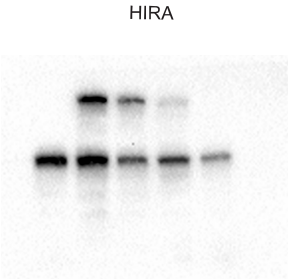

Supplement: S2 Data — (PDF) [file pgen.1011711.s016.pdf]
