# Supplementary material for: Ubinuclein 2 is essential for mouse development and functions in X chromosome inactivation
Source: PLoS Genet. 2025 Jun 2;21(6):e1011711. doi: 10.1371/journal.pgen.1011711 (PMC12165345; doi:10.1371/journal.pgen.1011711)
Supplement: Attachment — Submitted filename: Point_to_point_response.pdf [file pgen.1011711.s021.pdf]

Response to the comments from Reviewer #1

In this manuscript Monfort and colleagues study the HIRA complex cofactors Ubn1 and Ubn2, finding that mutation of Ubn2 but not Ubn1 causes partially penetrant embryonic lethality. Ubn1/2 double mutation causes fully penetrant embryonic lethality, with increased developmental arrest in females. The authors find that Ubn1/2 and Hira leads to impaired gene silencing by XCI and H3K27me3 deposition.

These findings are of very high interest to the XCI community and will warrant publication in PLoS genetics. The implication, although not explored here, that H3.3 may play a role in XCI is particularly enticing. However, many of the experiments presented are not fully quantified with appropriate statistical analysis, which will need to be performed.

Response: We thank the reviewer for finding our study of high interest and the insightful comments that have helped to further improve our manuscript.

Major comments

1. More quantitation of the data should be performed, together with appropriate statistical testing for data where conclusions are drawn, particularly for colony counting and IF experiments. The claim that XCI failure is greater in the Ubn1/2 dko than the single ko's appears valid by eye from the RNAseq, but this should also be tested statistically.

Response: We have addressed the reviewers request by adding new experiments and statistical testing in our revised version. In particular we now present extensive analysis of Xist localization and recruitment of histone modifications in Ubn1/Ubn2 double mutant ESCs and test the statistical difference to WT controls after Xist induction in revised Figure 6. We also have performed statistical testing for Xist mediated X-linked gene repression in all genotypes and present our analysis in new Figure 5F. This analysis includes all X-linked genes that are repressed to 0.6 fold or less in WT controls and is plotted as mean fold-change with errorbars indicating standard deviation. A paired 2-sided t-test was used and is highly significant for all comparisons. The distribution of effects of Xist on X-linked and autosomal gene expression is shown in Fig. 5Dand G. Related to this, we have also corrected a mistake in the y-axis labels in panel D as pointed out by reviewer 2 (minor point d). From this analysis we conclude that the effect on gene repression is a statistically significant between all genotypes. We would like to note that performing the analysis by determining gene expression changes with DESeq2 and comparing the fold-change values of all genes that are repressed to 0.6 or less fold-change in each genotype using a unpaired 2-sided t-test assuming equal variance we get the same result with the exception that the difference between Hira and Ubn2 mutant ESCs is no longer statistical significant. We provide this analysis at https://github.com/WutzLab along with all Python and R scripts (in RNAseq/DESeq2/Dox_vs_noDox/Analysis_Dox_vs_Ctrl.xlsx)

2. The Fig5 legend says that a p-value cut off was used for DE genes in the RNAseq. This would be inappropriate as it implies no correction for multiple testing. Please clarify whether multiple testing was performed, and if not, present only multiple testing corrected results.

Response: We thank the reviewer for raising this issue. Indeed we analyzed our RNAseq dataset with 2-sided p-values assuming equal variance. Although, we agree that multiple testing predisposes to a rather large false discovery rate, we selected genes with a large difference of means. It can be expected that the false discovery rate is not cumulatively increased by such a selection and hence the mathematical criteria of a p-value cutoff and biological relevance of at least 2-fold change are appealing to us for making a gene list (mainly as the meanings of statistical and biological measures are preserved). The reviewer's comment led us to explore Bonferroni correction and Benjamini-Hochberg critical values to attain a defined false discovery rate. However, both methods cause the elimination of nearly all genes likely due to the large number of comparisons. In the revised version we therefore turned to a different approach and reanalyzed differential gene expression between all genotypes using DESeq2. Our new analysis is shown in new Figure 5A, Suppl. Figure 4C, and Suppl. Table 4. DESeq2 uses log transformed read count data and makes several statistical assumptions for sample normalization and statistical testing. DESeq2 adjusted p-values are lower than our original p-values before adjusting for multiple testing, which allows for discovery of a greater number of differentially expressed genes. We have therefore included in Supplementary Table S4 all significantly differentially regulated genes together with statistical parameters provided by DESeq2. In addition, we list separately genes that are 1.5 and 2 fold up- and down-regulated for all genotypes against WT. From our new analysis we conclude that Ubn1 and Ubn2 single mutant cells have minimal expression changes. Consistent with our original analysis the Ubn1/2 double mutation has the greatest effect, but overlap of up- or down-regulated genes with Hira mutant cells remains limited.

3. What is the explanation for there being no overlap in DE genes between the different mutants? This would seem to contradict the claim that these proteins are in complex together. Genomic experiments are performed 48 hours after Xist induction, however it is not clear when the cells begin to die. Is it possible that the RNAseq readout is clouded by cells in the process of dying?

Response: We thank the reviewer for the comment. Our differential gene expression analysis between different genotype and WT considered only samples without Xist treatment. Therefore we can rule out that effects of Xist on cell viability and overlap of differential gene expression. We have clarified this in the text on page 11.

Following the remark of reviewer 2 (point 5) we have also analyzed the X chromosome number in WT and Ubn1/2 double mutant cells (new Supplementary Figure 3D). This analysis indicates that during generation of the double mutation ESCs have lost the second X chromosome and the representation of X-linked genes in the downregulated group might explain some of this effect. However, even considering only up-regulated genes only a minority overlaps with downregulated genes in Hira mutant cells. As we have used 3 replicates for each genotype we suggest that there is a biologically different effect of loss of Hira and of both Ubinuclein genes, which is surprising as HIRA protein seems drastically reduced in Ubn1/2 double mutant ESCs (new Figure 5C). Although we were able to detect more differentially regulated genes using

DESeq2, overall our new analysis is compatible with our earlier conclusions and demonstrates that effects of both mutations on gene expression are relatively modest with approximately 150-200 and 450-530 2-fold up or down regulated genes in Hira and Ubn1/2 double mutant ESCs. For the mutations in individual Ubinuclein genes there are hardly any changes that can be considered biologically relevant.

One explanation for the limited overlap of expression changes might be the absence of large effects of mutations on gene expression, which might amplify subtle changes that might originate from clonal variation (included the loss of the second X chromosome). This idea is consistent with earlier analysis of Hira mutant ESCs that also has noted only subtle effects on gene expression (Goldberg et al. 2010). We believe that the near absence of HIRA protein in Ubn1/2 double mutant ESCs indicates a substantial loss of HIRA complex function. Biological differences might arise from presence of either Ubinuclein or HIRA proteins before degradation, which might differentially affect the histone H3.3 pool. However, considering the limited amount of detectable protein, it appears that such differential effects are likely subtle.

4. The text says that Xist foci were the same between mutants, however the FISH data in Fig6a appears to show greatly reduced Xist localisation in the mutants. Have I misinterpreted something? If not, I think foci volume should be quantified.

Response: We thank the reviewer for the comment. In response, we have exchanged the images in Figure 6 and reanalyzed Xist expression in Ubn1/2 double mutant ESCs and present the new data in revised Figure 6B-F. Our analysis shows that Xist clusters in the double mutant ESCs reaches the size of control WT ESCs. However, we note a reduced number of cells with the largest cluster size. We interpret these observations as follows. Xist expression is efficient in Ubn1/2 double mutant ESCs. However, there are likely small differences in cluster formation that might be caused either by active genes due to inefficient gene repression by Xist or impaired chromatin modifications. Since the maximal cluster sizes are very comparable between the control and Ubinuclein mutant ESCs we believe that Xist induction is not materially affected, which is further consistent with our RT-PCR and RNAseq analysis.

Minor comments

1. It wasn’t clear to me if the HATX cells were female, male or haploid. Please clarify.

Response: We thank the reviewer for the comment and include a detailed description of our cell system in the revised version on page 9. Initially HATX ESCs were derived as haploid embryonic stem cell lines as described in Monfort et al., 2015. During culture haploid ESCs gain a diploid genome likely by aborting cytokinesis and we used the cells after diploidization as homozygous diploid ESCs, which possess two TX X chromosomes from which Xist can be induced. During culture of mouse ESCs one of the two X chromosomes is often lost. We also observe loss of the second X chromosomes in our cells. Our inducible system allows analysis of relative changes before and after Xist induction with Doxycycline, whereby the number of X chromosomes remains the same in each clone. However, there are two experiments where we perform comparisons between different ESC clones. Firstly, the heatmap in Figure 6E shows all

ESC lines with and without Xist induction separately. We have in this case computationally compensated for the different number of X chromosomes using the sum of TPM values for each clone before Xist induction. Secondly, the ATACseq experiment in Suppl. Fig. 7 shows chromatin accessibility (ATACseq read coverage) over the X chromosome and autosomes separately. In this case less coverage is observed in the double mutant cells compared to WT. The Ubn1/2 double mutant ESCs had been extensively engineered and we suspect that during the repeated selection for homozygous UBN2-HA, ΔUBN1, and lastly ΔUBN2-HA the second X chromosome had been lost, whereas parental ESCs maintained two X chromosomes in a proportion of the cells. We show an analysis of X chromosome number in the revised version in Supplementary Fig. 3D and have included a description of our cell system in the text on page 9 and consider the potential effects on our analysis on page 11, and page 15.

2. The ordering references to Fig4 were incorrect in the text.

Response: We have corrected our mistake in the revised version.

3. There is a typo in this sentence – “Xist forms a repressive compartment over non-genic regions of the X chromosome.” It should read “genic” regions

Response: We thank the reviewer for pointing out a need for a better introduction and explanation. Xist initially appears to affect regions on the X chromosome that do not harbor genes and primarily reside in the center of the Xi chromosome territory. Genes only associate with the Xist cluster after they have become repressed (Chaumeil et al., 2006). This observation has led to the idea of a "repressive compartment" over non-genic regions of the X and suggested a partition of Xi chromatin. The repressive compartment over non-genic regions has been proposed to function as a platform for gene repression. The study also shows that a mutant version of Xist that is defective in gene repression still leads to the formation of a repressive compartment with a number of Xi characteristic histone modifications, but genes would then not associate with the Xist domain but remain in the periphery of the chromosome territory. We have extended the introduction on page 5, where we cite the study by Chaumeil et al.

Response to the comments from Reviewer #2 (orig. uploaded as an attachment):

In this manuscript, Monfort et al. reported a female-specific role of the HIRA complex on *Xist*-mediated X inactivation (XCI) using engineered mutations in in vivo and in vitro mouse models. The in vivo model clearly showed a female-biased defect in early embryogenesis when *Ubn2, Ubn1*&2, or *Hira* but not *Ubn1* was mutated. The authors suggest that UBN2 and HIRA play a major role while UBN1 has a minor role in H3.3 deposition, which is essential for mouse embryogenesis with a more severe defect in female embryos. By using *Xist*-inducible haploid ESC lines, the authors confirmed the HIRA complex affects XCI as reported from their early study in 2015. Furthermore, they examined how the process of XCI was affected and found the establishment of H3K27me3 on the Xi was impaired by *Ubn1/Ubn2* double mutations, which provides new insights of the HIRA complex function in XCI and supports the results from their in vivo mouse models. Overall, this manuscript is well written, and the findings are supported by the data, though how the HIRA complex affects PRC2 mediated enrichment of H3K27me3 on the Xi has not yet been explored. Several points below could be clarified to strength this study.

Response: We thank the reviewer for finding our manuscript well written and the comments that have allowed us to further improve our study and strengthen our conclusions.

1. The mutant embryos were examined at E10.5-12.5. The authors should explain why not examine earlier stages, e.g., E5.5-8.5 when random XCI occurs. In addition, is there any phenotype in placenta?

Response: We have analyzed embryos at different stages and then focused on E10.5 - E12.5 for two reasons. Firstly, we expected a phenotype difference between male and female embryos might arise starting at E7.5 as this is the time point at which Xist mutant female embryos arrest (Marahrens et al, 1997). For Ubn2 and Ubn1/2 double mutant embryos a partial defect in Xi silencing would be expected, which could lead to a milder phenotype. By selecting E10.5 we anticipated to observe greater morphologic differences between male and female mutant embryos. Although, we have recovered embryos of both sexes the time of death of mutant embryos appeared to show substantial variability. This was paralleled with morphological and anatomical differences. Variable phenotypes are also observed for the Hira mutation (Roberts et. al., 2002). This study also investigated placental development in detail and reports a defect in chorioallantoic fusion in two thirds of all mutant embryos. In our revised version we provide images of placentae from a litter of E12.5 male and female embryos of a cross of Ubn2-/- males with Ubn2+/- females. This analysis shows that placental development proceeds at least in some of the Ubn2 mutant males and females. However, we cannot rule out that a placental defect contributes to loss of some embryos at earlier stages. For the Ubn1/2 double mutation we anticipate a placental component of the phenotype and show now that HIRA protein is drastically reduced in double mutant ESCs (Fig. 5C). In Ubn1 and Ubn2 mutant mice HIRA protein is reduced compared to WT, but clearly present (Fig. 5B).

2. The authors examined *Xist* cluster, H3K27me3 enrichment and H4K16ac depletion on the Xi (Fig 2D-F) in MEFs from *Ubn2* heterozygous and homozygous mutant embryos and show no

effects. It is better to have nuclei counts along with these pictures. No effect on H3K27me3 in *Ubn2* mutant MEFs is different from the in vitro ESC study. The authors should discuss this discrepancy. In addition, omics analyses such as RNA-seq and/or ChIP-seq of H3K27me3 in mutant MEFs could be helpful to examine molecular changes in embryos in comparison to the in vitro system.

Response: We thank the reviewer for the comment and would like to respond as follows. We anticipated that the female homozygous Ubn2 mutant embryos that could give rise to MEF lines had established XCI. This is consistent with the partial defect of Xist function in Ubn2 mutant ESCs. Our analysis was mainly focused to examine Xist expression to rule out a defect in XCI choice and the possibility that surviving female Ubn2 mutant embryos might have lost the second X chromosome. We believe our qualitative data confirming Xist expression can be informative as to show that surviving embryos have established XCI.

3. In the Xist survival assay, Ubn1-/- showed a minor increase of survival and Ubn1-/-Ubn2+/- showed a strong survival similar to Ubn2-/-, suggesting an effect on XCI. However, no phenotype was observed in in vivo mouse model for Ubn1-/- or Ubn1-/-Ubn2+/-. This difference should be discussed. Could it be due that the fast inducible Xist system is sensitive to the UBN dosage?

Response: We thank the reviewer for pointing out a need for clarification of our nomenclature. We realized that the + in the description of the Ubn1-/-Ubn2+/- ESCs is misleading as these ESCs are heterozygous for a complete and a partial deletion of Ubn2. The PCR in Figure 4K shows that the left flank of the intended Ubn2 deletion is absent and only the right primer pair generates a product demonstrating that the N-terminal part is deleted from the second allele. Initially, we have included this ESC line in our experiments as we thought it be a homozygous mutant for Ubn2 based on the left PCR. However, Western blot (Fig. 4L) clearly shows UBN2 protein is detected, although at a lower level and possibly lower molecular weight. The effect on Xist mediated gene repression is comparable to Ubn1/2 double mutant ESCs (see Response Figure 2 below, Ubn1_2 indicates Ubn1-/-Ubn2+/- ESCs, and Ubn1/2 and double mutant ESCs). We suggest that in the Ubn1-/-Ubn2+/- ESCs, remaining UBN2 protein might be impaired by its reduced abundance or possibly lacking domains that are important for interaction with H3.3 and HIRA and located in the on the N-terminal part of the deletion. The later is consistent with our observation that the left flanking PCR product is absent. We have added an explanation to the legend of Fig. 4KLM that the cells appear functionally equivalent to Ubn1 and Ubn2 double mutant cells as judged by the effect on *Xist* mediated X-linked gene repression and changed the naming to ΔUbn1/ΔUbn2* to indicate that this is ESC line is heterozygous for our full and partial Ubn2 deletion.

4. In Fig 4L, the western blots seem to show that HIRA protein levels were reduced by *Ubn* mutants, with the most effect in the double mutants. Is it possible that UBN stabilize HIRA by forming the complex given that *Hira* expression was not affected? This needs to be discussed.

Response: We thank the reviewer for bringing this interesting point to our attention. Interdependence of HIRA complex proteins has been previously reported (Bouvier et al., 2021, and Xiong et al., 2018). We have addressed the reviewers request by analyzing HIRA protein in Ubn1 and Ubn2 mutant mice and Ubn1/2 double mutant ESCs. We show the new Western

analysis in Fig 4B and C. Mutation of either Ubinuclein gene in mice reduces HIRA protein, whereby we would estimate that the abundance is halved. In Ubn1/ double mutant ESCs HIRA protein is dramatically reduced (Fig. 5C). Notably, this near loss of protein is paralleled with unchanged Hira transcript levels in our RNAseq analysis. Our data suggest that the abundance of HIRA is regulated by both UBN1 and UBN2. We have added this observation in the text of page 11 (end of the first paragraph).

5. Interestingly, it seems that downregulated but not upregulated genes in *Ubn* double mutants or *Hira* mutants compared to WT ESCs are enriched in X-linked genes (Supp. Table 4). This is further supported by reduced chromatin accessibility for the X in *Ubn* double mutants vs. WT in NT while similar accessibility was on chromosome 1 (see Supp. Fig 7A, B). Does this suggest the role of the HIRA complex in upregulation of the X chromosome? This should be described. In addition, the chromosome location and expression fold changes and significance should be included for DE genes. It is also better to have a complete table of the transcriptome and changes before and after Xist induction in WT and mutants.

Response: We thank the reviewer for the important observation, which has led us to investigate a likely reason. Based on additional analysis of X chromosome numbers in WT and Ubn1/2 double mutant ESCs (new Suppl. Fig. 3D), we suggest that the overrepresentation of X-linked genes among downregulated genes in Ubn1/Ubn2 double mutant ESCs is likely caused by the loss of the second X chromosome.

The HATX ESCs used in our study were derived as haploid embryonic stem cell lines as described in Monfort et al., 2015, but had become diploid over time. We used the as homozygous diploid ESCs, which possess two TX X chromosomes from which Xist can be induced. During culture of female mouse ESCs loss of one of the two X chromosomes often leads to accumulation of XO cells. We also observe loss of the second X chromosomes in our cells. Our inducible system allows analysis of relative changes before and after Xist induction with Doxycycline, whereby the number of X chromosomes remains the same in each clone. However, there are two experiments where we perform comparisons between different ESC clones. Firstly, the heatmap in Figure 6E shows all ESC lines with and without Xist induction separately. We have in this case computationally compensated for the different number of X chromosomes using the sum of TPM values for each clone before Xist induction. Secondly, the ATACseq experiment in Suppl. Fig. 7 shows chromatin accessibility (ATACseq read coverage) over the X chromosome and autosomes separately. In this case less coverage as the reviewer correctly points out is observed in the double mutant cells compared to WT. The Ubn1/2 double mutant ESCs had been extensively engineered and we suspect that during the repeated selection for homozygous UBN2-HA, ΔUBN1, and lastly ΔUBN2-HA the second X chromosome had been lost, whereas parental ESCs maintained two X chromosomes in a proportion of the cells. We show an analysis of X chromosome number in the revised version in Suppl. Fig. 3D and have included a description of our cell system in the text on page 9 and consider the potential effects on our analysis on page 11, and page 15. From our analysis of Hira mutant ESCs we believe that a role for the HIRA complex in X chromosome upregulation is unlikely.

6. Lack of detailed descriptions of the analyzing process of omics data in Methods, e.g., the method to data normalization and call DE genes, ChIP and ATAC-seq peaks.

Response: We have addressed the reviewers request by adding a description of our computational analysis strategy to the methods section on page 23. All Python and R Scripts that were used are now available at https://github.com/WutzLab. To facilitate reproducing our analysis, we also provide genome annotation and intermediate data. Genomic coverage wiggle files for ChIPseq and ATACseq can be downloaded using a link provided in the README.md file of the github repository.

Minor points:

a) it is better to mark neural tube closure defects in supp Fig 1;

Response: We have followed the reviewers suggestion and added a red arrow to point to the exencephaly in revised Supplementary Figure 1A. In addition, we have rephrased the legend to be clear that the observation on exencephaly led us to infer an anterior neural tube closure defect. There is no indication of neural tube closure defects in the posterior region of the embryos.

b) Page 9, line 10, Supp Fig 2B-D is the results of genomic PCR and RT-PCR not western analysis;

Response: We have thank the reviewer for the comment and have changed the text to say that the Western analysis is shown in Figure 4B, genomic PCR and RT-PCR in Suppl. Fig. 2B-D in the revised version.

c) Page 9, line 4 from the bottom, Fig 1G should be 4G;

Response: We have corrected the figure reference in the revised version.

d) Fig 5D showed plots similar to supp Fig 4D with different y-axis (expression fold change +Dox/-Dox) and the legends are almost same. Please clarify the difference;

Response: We thank the reviewer for pointing out our error in y-axis labels that has occurred when composing the figure. We have corrected this mistake and replaced Fig. 4D in the revised version. The original plots are also included in Response Figure 2 below. Suppl. Fig. 4D was plotted correctly and shows the same analysis but includes Xist, which is strongly upregulated. Hence, the effects on X-linked genes are compressed on the y-axis. Our intention was to make the effect of Xist on X-linked genes easier to see in Fig. 4D.

d) in Fig 4H, no description for FT, in 4J, K, why is there a band specific for deletion in + cells?

Response: We have included the missing details in the legends of Figure 4 panels H, J, and K. The positive controls (+, and C+) in J and K are a heterozygous clones which show PCR products for wild type and deletion alleles.

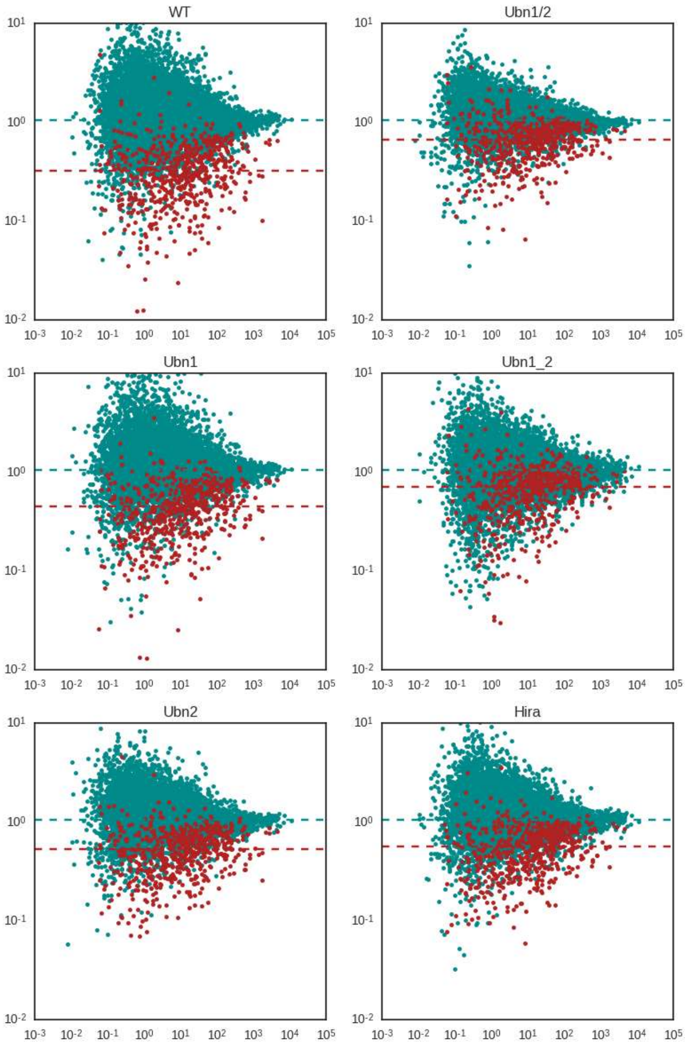

Response Figure 2 - X-linked gene repression by *Xist* induction. Plot showing fold change of expression after *Xist* induction with Doxycycline for 48 hours (+Dox/-Dox TPM ratio) of X-linked (red) and autosomal (green) genes (y-axis) against gene expression level in WT control ESCs (TPM) on x- and y-axis. Combined triplicates are shown for WT, *Ubn1*, *Ubn2*, and *Ubn1/Ubn2* (Ubn1/2) mutant ESCs. A single replicate is shown for one Ubn1/Ubn2 double mutant ESC clone that had lost the left but retained the right flanking region of the ΔUbn2 deletion (Ubn1_2). For this clone a similar defect in Xist mediated X-linked gene repression is observed as in *Ubn1/Ubn2* double mutant ESCs suggesting that the partial deletion causes a severe impact on UBN2 function likely by removing the N-terminal histone H3.3 and HIRA interaction region. Dashed lines mark no expression change (green) and the mean expression change for X-linked genes (red). Data are TPM normalized to autosomes therefore the green dashed line corresponds to the mean expression change of autosomal genes.

Response to comments from Reviewer #3:

In this manuscript, Monfort et al. investigate role of the HIRA complex including UBN1/UBN2 in X chromosome inactivation (XCI). They demonstrated that KO mice of Ubn1/Ubn2 or Hira have severe female-specific embryonic lethality, suggesting their important roles in XCI in vivo. They also showed that the mutation of Ubn1/Ubn2 or Hira in mouse ESCs compromises Xist-mediated gene silencing on the inactive X chromosome (Xi). Mechanistically, H3K27me3 accumulation on the Xi upon Xist induction is impaired in Ubn1/Ubn2 or Hira mutant ESCs.

I think the in vivo KO mouse data is straightforward and convincing for their conclusions. My concern is, however, there are several points still preliminary or unclear for me and these points should be clearly answered to improve this study.

Response: We thank the reviewer for finding our analysis of Ubinucleins in mice convincing and the helpful suggestions for improving our study. We have addressed the reviewers concerns by adding additional experiments, improving the presentation of our data, and making clarifications in the text in the revised version.

Major concerns

1. Although the authors described "The percentage of nuclei showing focal domains of JARID2, and H3K27me3 overlapping with Xist was slightly lower in ΔUbn2 and ΔHira than control ESCs after 24 hours of Xist induction, whereas H2AK119ub1 remained unchanged (Fig. 6AC and Suppl. Fig. 5B-E)." on page 12, I can't seem to find the images of H2AK119ub1 staining. As the Brockdorff lab previously showed the presence of Xist-PRC1-PRC2 cascade in XCI initiation (Almeida et al 2017), I think these staining images of H3K27me3 and H2AK119ub1 are important and should be displayed in a main figure. In addition, several images such as RING1B staining in Fig 6 and Suppl Fig 5 lack quantification. It is necessary to show quantification charts for all of the imaging data with statistic evaluation. The number of counted cells should also be indicated.

Response: We thank the reviewer for the suggestions. In response we have restructured Fig. 6 to focus on ΔUbn1/ΔUbn2 ESCs. We show new images of Xist (Fig. 6AB), H3K27me3 and H2AK119Ub along with statistical testing (Fig. 6C-F). Our analysis indicates highly significant differences in H3K27me3 and H2AK119ub1 between WT control and ΔUbn1/ΔUbn2 ESCs. For Jarid2 and Ezh2 the difference does not reach significance but a trend consistent with the histone marks can be observed (Fig. 6GH). We included this new analysis in the text on page 12 and 13. All numerical data is included in the excel formatted file Source_Data_Fig_6.xlsx.

2. Relating to 1, H3K27me3 ChIP-seq was performed with ΔUbn1/ΔUbn2 ESCs (Fig 7), but H3K27me3 immunostaining was performed with ΔUbn2 and ΔHira ESCs (Suppl. Fig. 5BCDE), not with ΔUbn1/ΔUbn2 ESCs. Is it possible to show an image and quantification data about H3K27me3 in ΔUbn1/ΔUbn2 ESCs? I think this data is important to support the idea that overall H3K27me3 accumulation (IF data) is only partially impaired by the HIRA complex mutations but

local H3K27me3 accumulation (ChIP-seq) is strongly attenuated in these mutant ESCs by comparing IF and ChIP-seq in the same genetic background.

Response: We agree with the reviewer that the ∆Ubn1/∆Ubn2 ESCs are most important for our analysis. We have therefore performed H3K27me3 and H2AK119ub1 immunofluorescence staining and focused Fig. 6 to include this new data along with a description in the text on page 12.

3. By using ChIP-seq analysis, the authors showed that H3K27me3 accumulation upon Xist induction is impaired in ∆Ubn1/∆Ubn2 or ∆Hira ESCs at around TSS and gene body (Figure 7A). However, immunostaining of EZH2/H3K27me3 indicated the accumulation of PRC2/H3K27me3 on the Xi is only partially impaired in ∆Ubn2 and ∆Hira ESCs (Suppl. Fig. 5BCDE). These data suggest that Xi chromatin change in the HIRA complex mutants is not X chromosome-wide. Rather, it might be limited to certain genomic regions on the Xi. My concern is that the HIRA complex mutants would have a lack of H3.3 incorporation at limited regions such as around TSS and gene body on the Xi, which affects H3K27me3 accumulation on the Xi partially. Therefore, I think it is important to show whether H3.3 is actually incorporated into the Xi chromatin upon Xist induction in initiation of XCI and how much of them is inhibited in the HIRA complex mutant ESCs. This experiment will clarify the molecular mechanism of HIRA-dependent XCI regulation. Is it possible to examine H3.3 ChIP-seq and immunostaining with an anti-H3.3 antibody in Xist-inducible ESCs?

Response: We agree with the reviewer that the H3K27me3 is affected over genes but not abrogated entirely as we observe a focal enrichment after *Xist* induction. Considering the function of the HIRA complex for incorporation of histone H3.3 specifically at genes but not pericentric and telomeric heterochromatin a local effect over genes could be explained. To address the reviewers point if histone H3.3 is enriched after Xist induction, we have performed two experiments. We have attempted to perform histone H3.3 CUT and RUN but did not obtain useful results likely due to limited specificity of the antibody for H3.3 over canonical H3 resulting in a wide and nearly random read distribution. To address the question if histone H3.3 might be enriched in Xi chromatin we have performed immunofluorescence staining with H3.3 antibodies and an HA-tagged histone H3.3 (Response Figure 2, below). In these experiments no focal enrichment could be observed after Xist induction . From this observation we suggest that histone H3.3 and the HIRA complex may play a role in the initial phase of repressing genes, when these are still active. Histone H3.3 deposition and HIRA function are likely facilitating a change of the expression state but are not further involved in repression once genes have been silenced. We also would like to point out that previous studies have implicating Atrx in the maintenance phase of XCI (Sarma et al., 2014, and Baumann et al., 2009). In addition, the Atrx mutation has been studied in mice (Garrick et al., 20026), and H3.3 ChIP has been reported previously (Goldberg et al., 2010, Elsässer et al., 2015, and Xiong et al., 2018). However, none of these studies have obtained any evidence for enrichment of histone H3.3 over the Xi. Although, we suggest that the HIRA complex and histone H3.3 function at the transition to gene silencing, we do not wish to rule out that enrichment of histone H3.3 might have been missed for technical reasons. Additional experiments and new cell lines will be required to fully resolve this question.

A

B

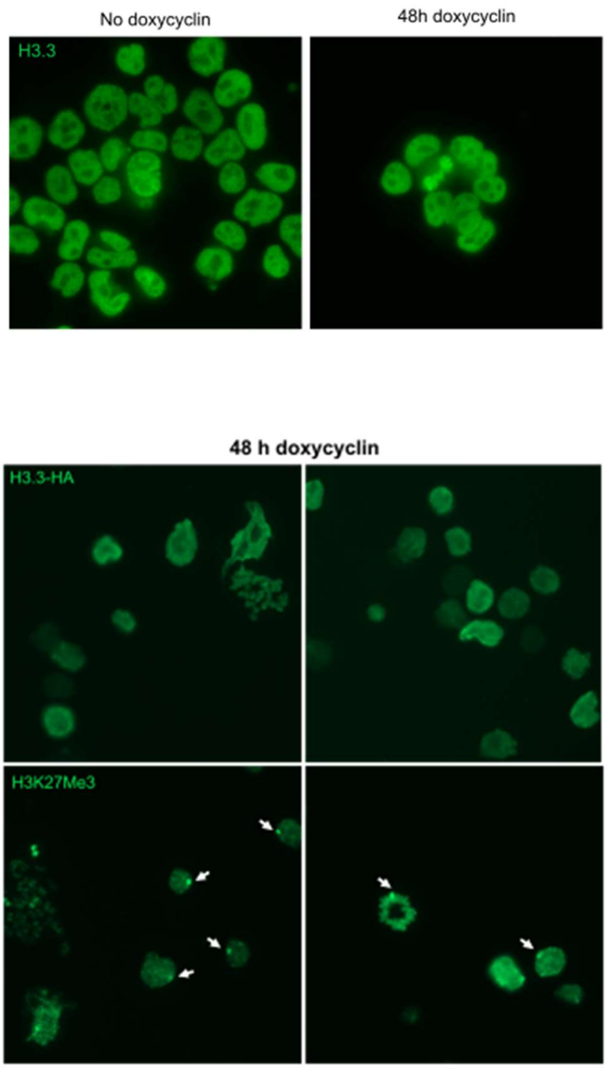

Response Figure 2 - Histone H3.3 localization. (**A**) Immunofluorescence image (above) using an antibody specific for H3.3 does not reveal a focal enrichment of H3.3 after *Xist* induction for 48h with doxycycline. (**B**) HATX ESCs carrying a stably integrated piggyBac transposon vector for expression of HA-tagged histone H3.3 were stained with an antibodies specific for HA (top) and H3K27Me3 (bottom) after 48h of Xist induction with doxycycline. Focal enrichment of H3K27Me3 (arrows), no enrichment was observed with the HA antibody.

4. A sentence “ATAC-Seq profiles showed that accessibility was similar in WT and ΔUbn1/ΔUbn2 ESCs before Xist induction.” on page 15 would be misleading as the X chromosome-wide ATAC-seq data shown in Supple. Fig, 7A appears a clear decrease of ATAC-seq peaks in ΔUbn1/ΔUbn2 ESCs even before Xist induction.

Response: We thank the reviewer for the observation. A similar point was raised by reviewer 2 (point 5) and led us to investigate the reason. We performed X chromosome painting analysis and identified that the X chromosome numbers in WT and Ubn1/2 double mutant ESCs differ (new Suppl. Fig. 3D). We suggest that the decrease of X-linked read coverage in the ATACseq experiment in Ubn1/Ubn2 double mutant ESCs is likely caused by the loss of the second X chromosome. The HATX ESCs used in our study were derived as haploid embryonic stem cell lines as described in Monfort et al., 2015, but had become diploid over time. We used the as homozygous diploid ESCs, which possess two TX X chromosomes from which Xist can be induced. During culture of female mouse ESCs loss of one of the two X chromosomes often leads to accumulation of XO cells. We also observe loss of the second X chromosomes in our cells. Our inducible system allows analysis of relative changes before and after Xist induction with Doxycycline, whereby the number of X chromosomes remains the same in each clone. However, there are two experiments where we perform comparisons between different ESC clones. Firstly, the heatmap in Figure 6E shows all ESC lines with and without Xist induction separately. We have in this case computationally compensated for the different number of X chromosomes using the sum of TPM values for each clone before Xist induction. Secondly, the ATACseq experiment in Suppl. Fig. 7 shows chromatin accessibility (ATACseq read coverage) over the X chromosome and autosomes separately. In this case less coverage as the reviewer correctly points out is observed in the double mutant cells compared to WT. The Ubn1/2 double mutant ESCs had been extensively engineered and we suspect that during the repeated selection for homozygous UBN2-HA, ΔUBN1, and lastly ΔUBN2-HA the second X chromosome had been lost, whereas parental ESCs maintained two X chromosomes in a proportion of the cells. We show an analysis of X chromosome number in the revised version in Suppl. Fig. 3D and have included an explanation for the reduced X-linked read coverage in Suppl. Fig. 7A in the revised text on page 15.

5. They conclude that insufficient H3K27me3 accumulation by Xist in HIRA complex mutant ESCs might be a cause of impaired silencing of X-linked genes in these ESCs. As they have H3K27me3 ChIP-seq data and RNA-seq data, is it possible to compare the degree of insufficient H3K27me3 accumulation and the level of impaired gene silencing for X-linked genes in the mutants? I think this comparison could further strengthen the role of H3K27me3 deposition in Xist-mediated gene silencing via Xist-HIRA axis.

Response: We thank the reviewer for the suggestion and have plotted the *Xist* mediated fold-change of H3K27me3 against the fold-change in gene repression for X-linked genes. We have added the new analysis in Suppl. Fig. 6B and a description to the text on page 13. From this analysis a strong trend can be observed that increase of H3K27me parallels reduction of gene expression in WT control ESCs. This trend is drastically reduced in ΔUbn1/ΔUbn2 ESCs consistent with our earlier conclusion. This new analysis shows that genes, whose repression by Xist is affected are also failing to acquire H3K27me3 at their TSS and gene body.

Minor concerns

1. Indication of genotypes in each figure should be in the same way. For example, some figures use ΔUbn1/Ubn2 and the other figures use ΔUbn1/ΔUbn2.

Response: We have changed the indication of the genotypes following the reviewers suggestion.

2. Similarly, Y-axis title is lacking in many charts. For example, all ChIP-seq data lack Y-axis title and need to be correctly indicated.

Response: We have added y-axis titles as requested.

3. Page 5

Edith Heard lab showed that only B repeat mutant is not sufficient to completely block the PRC1/2 accumulation on the Xi. Rather, they showed that both B and C repeats of Xist RNA is required for PRC1/2 recruitment on the Xi (Ref 29). This should be correctly described.

Response: We thank the reviewer for bringing this mistake to our attention. In the revised version, we now correctly state that Xist repeat B and C facilitate the recruitment of Polycomb complexes.

4. Figure 4CFM, Suppl Figures 2F&3AB

The charts in these figures present the result of colony forming assays with various ESC clones but are a bit confusing because two different forms of charts are used there. Is it possible to use the same form of chart in these figures?

Response: We have changed the presentation of the data from our assay and now use a consistent form for all experiments in each figure.

5. Suppl Figure 4AB

To help to recognize which genomic region is deleted in these clones, would it be possible to show the targeted region on the top of each map?

Response: We amended Suppl. Fig. 4A and B to indicate the position of the deletions.

6. Suppl Figure 5A

Images are too small to see the distribution of HIRA and UBN2. Enlarged images (ideally 2 or 3 cells per image) should be presented here.

Response: We have amended Suppl. Fig. 5 to show larger images and provide enlarged views in panel A.

7. The indication “Ubn2-HA ΔUbn1/ΔUbn2” is confusing and one can misunderstand these cells express exogeneous Ubn2-HA transgene. Can another name be used for this cell line? I think probably simply “ΔUbn1/ΔUbn2” or “ΔUbn1/ΔUbn2-HA” would be fine.

Response: We understand the reviewers concern and would like to respond in the following way. Generation of the Ubn2-HA ESCs is described on page 10 and makes clear that the HA tag was introduced into the endogenous Ubn2 gene. The following text describes derivatives ΔUbn1 Ubn2-HA ESCs before the ΔUbn1/ΔUbn2 Ubn2-HA. Although we find the reviewers suggestion appealing, we have maintained the nomenclature to be consistent among the ESC lines and in particular with the ΔUbn1 Ubn2-HA ESCs.

8. Pages 9 & 14

There are several typos. Fig. 1G on the page 9 and H3K4me4 on the page 14.

Response: We have corrected the wrong figure citation and the typo on page 14.
