# Supplementary material for: Ubinuclein 2 is essential for mouse development and functions in X chromosome inactivation
Source: PLoS Genet. 2025 Jun 2;21(6):e1011711. doi: 10.1371/journal.pgen.1011711 (PMC12165345; doi:10.1371/journal.pgen.1011711)
Supplement: Attachment — Submitted filename: Review report for Monfort et al 09 2024.docx [file pgen.1011711.s019.docx]

In this manuscript, Monfort et al. reported a female-specific role of the HIRA complex on *Xist*-mediated X inactivation (XCI) using engineered mutations in in vivo and in vitro mouse models. The in vivo model clearly showed a female-biased defect in early embryogenesis when *Ubn2,* *Ubn1*&2, or *Hira* but not *Ubn1* was mutated. The authors suggest that UBN2 and HIRA play a major role while UBN1 has a minor role in H3.3 deposition, which is essential for mouse embryogenesis with a more severe defect in female embryos. By using *Xist*-inducible haploid ESC lines, the authors confirmed the HIRA complex affects XCI as reported from their early study in 2015. Furthermore, they examined how the process of XCI was affected and found the establishment of H3K27me3 on the Xi was impaired by *Ubn1/Ubn2* double mutations, which provides new insights of the HIRA complex function in XCI and supports the results from their in vivo mouse models. Overall, this manuscript is well written, and the findings are supported by the data, though how the HIRA complex affects PRC2 mediated enrichment of H3K27me3 on the Xi has not yet been explored. Several points below could be clarified to strength this study.

1. The mutant embryos were examined at E10.5-12.5. The authors should explain why not examine earlier stages, e.g., E5.5-8.5 when random XCI occurs. In addition, is there any phenotype in placenta?

2. The authors examined *Xist* cluster, H3K27me3 enrichment and H4K16ac depletion on the Xi (Fig 2D-F) in MEFs from *Ubn2* heterozygous and homozygous mutant embryos and show no effects. It is better to have nuclei counts along with these pictures. No effect on H3K27me3 in *Ubn2* mutant MEFs is different from the in vitro ESC study. The authors should discuss this discrepancy. In addition, omics analyses such as RNA-seq and/or ChIP-seq of H3K27me3 in mutant MEFs could be helpful to examine molecular changes in embryos in comparison to the in vitro system.
3. In the Xist survival assay, Ubn1-/- showed a minor increase of survival and Ubn1-/-Ubn2+/- showed a strong survival similar to Ubn2-/-, suggesting an effect on XCI. However, no phenotype was observed in in vivo mouse model for Ubn1-/- or Ubn1-/-Ubn2+/-. This difference should be discussed. Could it be due that the fast inducible Xist system is sensitive to the UBN dosage?
4. In Fig 4L, the western blots seem to show that HIRA protein levels were reduced by *Ubn* mutants, with the most effect in the double mutants. Is it possible that UBN stabilize HIRA by forming the complex given that *Hira* expression was not affected? This needs to be discussed.
5. Interestingly, it seems that downregulated but not upregulated genes in *Ubn* double mutants or *Hira* mutants compared to WT ESCs are enriched in X-linked genes (Supp. Table 4). This is further supported by reduced chromatin accessibility for the X in *Ubn* double mutants vs. WT in NT while similar accessibility was on chromosome 1 (see Supp. Fig 7A, B). Does this suggest the role of the HIRA complex in upregulation of the X chromosome? This should be described. In addition, the chromosome location and expression fold changes and significance should be included for DE genes. It is also better to have a complete table of the transcriptome and changes before and after Xist induction in WT and mutants.
6. Lack of detailed descriptions of the analyzing process of omics data in Methods, e.g., the method to data normalization and call DE genes, ChIP and ATAC-seq peaks.

Minor points: a) it is better to mark neural tube closure defects in supp Fig 1; b) Page 9, line 10, Supp Fig 2B-D is the results of genomic PCR and RT-PCR not western analysis; c) Page 9, line 4 from the bottom, Fig 1G should be 4G; d) Fig 5D showed plots similar to supp Fig 4D with different y-axis (expression fold change +Dox/-Dox) and the legends are almost same. Please clarify the difference; d) in Fig 4H, no description for FT, in 4J, K, why is there a band specific for deletion in + cells?
